# Transferable, Living Data Sets for Predicting Global Minimum Energy Nanocluster Geometries

**DOI:** 10.1021/acs.jctc.4c00572

**Published:** 2024-07-24

**Authors:** Bart Klumpers, Emiel J. M. Hensen, Ivo A. W. Filot

**Affiliations:** Laboratory of Inorganic Materials and Catalysis, Department of Chemical Engineering and Chemistry, Eindhoven University of Technology, Eindhoven 5600 MB, The Netherlands

## Abstract

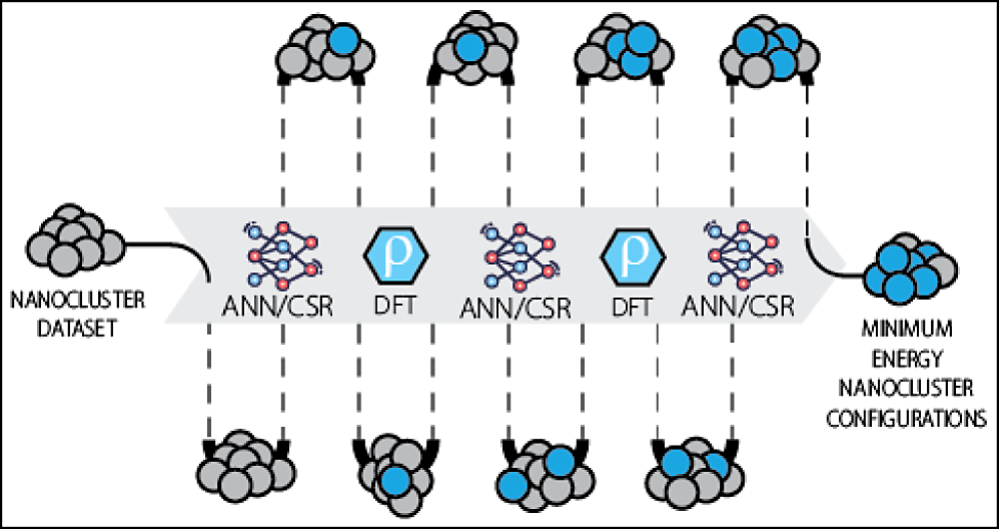

Modeling of nanocluster geometries is essential for studying the dependence of catalytic activity on the available active sites. In heterogeneous catalysis, the interfacial interaction of the support with the metal can result in modification of the structural and electronic properties of the clusters. To tackle the study of a diverse array of cluster shapes, data-driven methodologies are essential to circumvent prohibitive computational costs. At their core, these methods require large data sets in order to achieve the necessary accuracy to drive structural exploration. Given the similarity in binding character of the transition metals, cluster shapes encountered for various systems show a large amount of overlap. This overlap has been utilized to construct a living data set which may be carried over across multiple studies. Iterative refinement of this data set provides a low-cost pathway for initialization of cluster studies. It is shown that utilization of transferable structural information can reduce model construction costs by more than 90%. The benefits of this approach are particularly notable for alloy systems, which possess significantly larger configurational spaces compared to the pure-phase counterparts.

## 1 Introduction

Conventional heterogeneous catalysts are composed of an active metal deposited on a support material. The metal is deposited in the form of nanoclusters or nanoparticles: atomic aggregates ranging in size from tens to thousands of atoms. The size and shape of the particles determine the availability and chemical nature of the active sites where reagents bind. For small clusters, confinement results in localization of electrons on the metal atoms (compared to the bulk phase). Low coordination numbers for the atoms result in high propensity to form bonds with adsorbates, facilitating transfixation of otherwise weakly bound species as well as possible weakening of strong intermolecular bonds to promote chemical reactivity. As the cluster size increases, a gradual transition to bulk metal character takes place.^1,2^ For larger clusters, the diversity of atomic coordination environments increases. Site geometries for larger particles may lead to more favorable interaction with the molecular orbitals to activate a wider range of reaction intermediates.^3,4^ In order to understand the catalytic mechanism, it is thus important to know the nature of the metallic phase. Given their small size, experimental imaging of the clusters is oftentimes not possible, with theory being required to fill the gaps in knowledge.

Modeling nanocluster catalysis requires a model of the cluster. As there are many possible candidate geometries, direct numerical evaluation of all candidates is computationally intractable already for small clusters. The straightforward approach is therefore to utilize stochastic methods to limit the total number of samples.^5,6^ However, this introduces a risk-reward element: a larger number of samples increases the likelihood of encountering new stable structures but requires higher computational investment. In order to increase the value of the limited sample set, improved strategies propose informed selection of samples.^7,8^ One strategy utilizes the known free energy of particle facets or sites to predict stable structures.^9,10^ Genetic algorithms (GA) provide a generalization of this approach by proposing cluster geometries resembling stable structures encountered during the sampling.^11−13^ In recent years, statistical regression models have also been developed to tackle the abstract problem of relating cluster geometry and energy.^14−19^

A common motif remains with the methods, where after selection of samples it is still required to evaluate the energy properly. For catalytic application, this requires utilization of quantum chemical methods, making up the computationally most-costly step of the process. Surrogate models may be utilized to bypass the quantum chemical calculation by providing an alternative method for estimating the energy of clusters resembling known structures. Artificial neural networks (ANN) have been offered as one such model.^20−26^ The key to efficient utilization of ANN for this purpose lies in the quality of the data set, i.e., the set of known structures. This presents a dilemma as a (large) set of structures must first be generated before the ANN can be used. The common solution in ANN literature is to perform online learning.^23,25,27,28^ Stochastic sampling is performed initially by utilizing quantum chemical calculations, where this data is used to fit the ANN. The ANN may then be utilized as a surrogate for predicting the cluster energies. The ANN parametrization is updated throughout the structure exploration when new structural features are encountered.

As the ANN requires a data set containing at least several hundreds of structures in order to yield a reliable parametrization, an expensive quantum-chemical exploration remains necessary. In the current work, we propose to utilize transferable data sets as an alternative to stochastic initialization of the ANN data set. Comparing recent nanometal studies, the shape and structure of metal clusters appears to be similar among various metals, phases and supports.^13,14,23,29^ This may be rationalized as the metal atoms do not tend to form strongly directional bonds, instead forming various spherical stackings. A set of species-agnostic cluster geometries may therefore be utilized to sample the structure-energy relationship of the system of interest. Instead of reinitializing this data set for every study, information from earlier studies on other systems may be utilized to optimize the sampling. This is of particular interest where bi- or multimetallic systems are concerned, as dopants tend to have a comparatively minor effect on the cluster shape.

**Figure 1:**
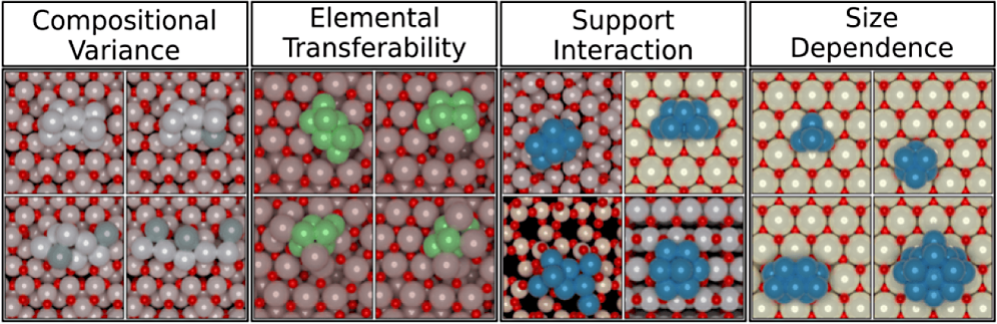
Overview of nanocluster systems studied using the transfer data set methodology. Cross-generational data set construction is investigated for 8-atom clusters with varying Pt:Sn compositions. Transfer to different alloys is investigated for NiIn. Support interactions and particle-size dependence are studied for pure metallics.

In this work, a discussion of the computational aspects of the transfer data set methodology is presented. Performance analysis shows a drastic decrease in both the computational cost and real-time execution of cluster-structural exploration. Several catalytic systems are analyzed to show broad applicability of the method, as illustrated in Figure 1.

- Initially, focus is placed on supported PtSn nanoclusters to showcase the benefits of the concurrent sampling of bimetallic compositions.
- Transferability of the data sets is shown by considering NiIn clusters.
- Dependence on the support structure is investigated by comparing pure metal clusters on Al_2_O_3_, CeO_2_, SiO_2_, and TiO_2_.
- Scale-up toward larger particles is discussed for the cobalt system.

## 2 Methods

ANN are used to map the energy-structure relationship of the nanoclusters, as obtained from density-functional theory (DFT). The description of these ANN is taken from earlier studies on adsorbate systems.^30^ In this respect, the clusters are themselves treated as polyatomic adsorbates. The ANN are then used to predict the energy for each atom in the cluster-support system:
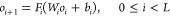
1

The architecture of the ANN is structured in layers, each consisting of a linear transformation defined by the matrix-vector pair () and a nonlinear transformation *F*_*i*_. Multiple layers may be connected to increase the complexity of the network. The input to subsequent layers is determined by the dimensionality of the coefficient matrix, denoted by the number of nodes. A separate ANN is constructed for each unique chemical element. This assures that the interactions are the same for each metal atom. Clusters with a varying number of atoms or different alloy compositions may be treated by combining the appropriate set of ANN.

In order for the ANN to map the correct energy to each of the clusters, network input is comprised of a basis set of functions sampling the structural or electronic environment of the atoms. These sampling functions are used to distinguish between different atomic environments in order to facilitate the assignment of atomic energies. The atomic energy decomposition is determined by the ANN itself by using a linear transformation for the final layer, thereby accounting for variations in the total number of atoms. The functional form of the basis functions was detailed in an earlier publication.^30^ The set of functions that is used to construct the ANN is determined using continuous similarity recurrence (CSR). This method allows removal of redundant functions and the identification of missing features in the basis set by considering the distribution of sampled values in the training data set. The architecture of the ANN is determined next by performing a hyperparameter screening of the layers, nodes and nonlinear functions. A penalty function is used to select the optimum, accounting for potential under- or overfitting.

The global minimum-energy structure of the clusters was obtained using ANN potentials coupled with a genetic algorithm (GA).^29,30^ The GA performs a sequence of manipulations of the cluster geometry. The energy of these geometries is evaluated, from which stable clusters may be identified. The GA iteratively creates new trial geometries by combining structural features from known stable clusters in an attempt to find the global minimum. A stochastic element of the method results in occasional inclusion of less-stable features in order to avoid local minima and to guarantee proper exploration of the configurational space of the clusters.

The computationally limiting step of the GA is the evaluation of the energy using quantum-chemical methods. ANN are employed to replace the expensive DFT calculations when deriving the cluster energies. The mathematical form of the ANN results in low computational cost. This allows evaluation of a large number of structures in the GA. Compared to other parametrized structure-energy models such as force-fields, ANN are capable of reproducing energies with very high accuracy when applied to a specific data set. Proper construction of this data set is therefore a key factor to consider. The flexibility of ANN allows them to learn complex relationships between data properties, but also results in unstable behavior in the extrapolation regime. Evaluation of the ANN input readily allows identification of this extrapolation regime, providing a detection method to avoid bad data from entering the GA.

**Figure 2:**
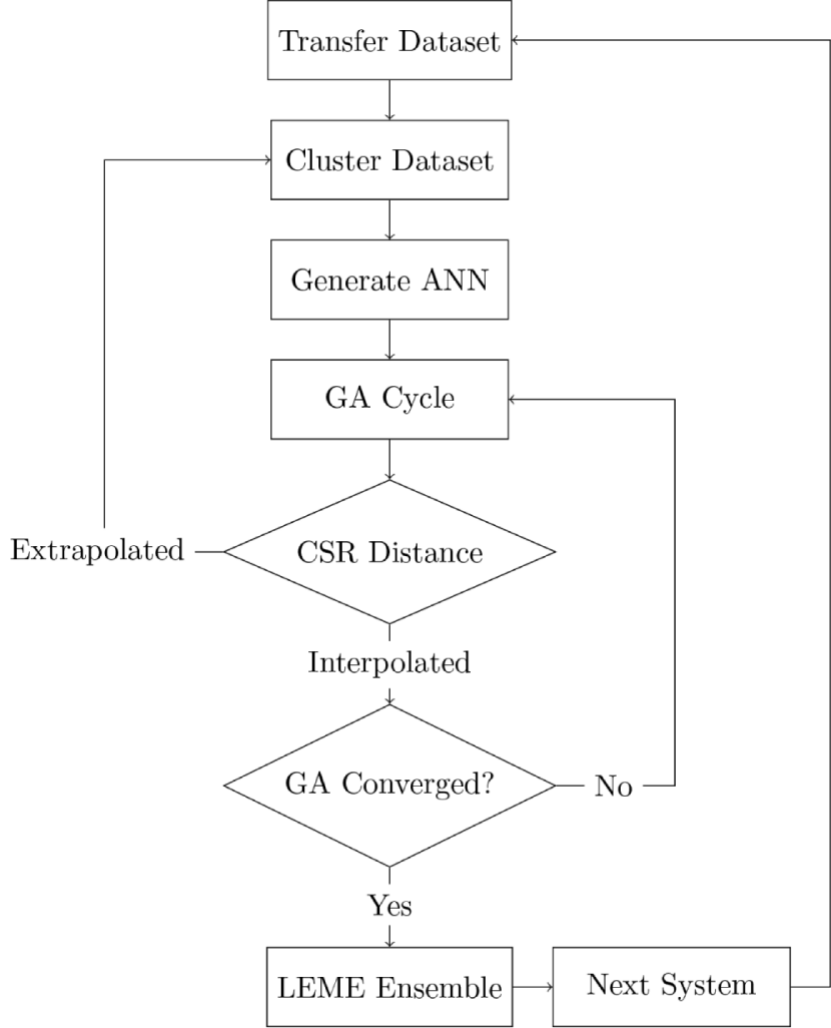
Outline of the transfer data set strategy for ANN-driven GA exploration of cluster LEME.

Proper fitting of the ANN typically requires large data sets. This would entail generation of a large number of cluster configurations, at which point there would likely no longer be any need to conduct further GA exploration. To circumvent this hurdle, the similarity between cluster geometries of various metals may be leveraged to construct a global data set of cluster geometries. An overview of this process is shown in Figure 2. Cluster data, obtained from earlier studies employing DFT-driven GA, was used to initialize the data set. When the data set is applied to study a new system, the cluster atoms are replaced by those of the appropriate metal. The difference in bulk lattice volume may be used to scale the interatomic bond distances. The clusters are then adsorbed onto the chosen support by utilizing a surface geometric potential^30^ to account for surface morphology. When an alloy is considered, random substitution of the metal atoms is performed to generate trial clusters with the desired composition. Due to the coexistence of various bulk alloy phases, volumetric scaling of the samples was performed using the largest of the pure-phase constituent volumes, thereby avoiding generation of high-stress states. The energies of the resulting set of geometries are evaluated using single-point DFT calculations. This yields a significant reduction in computational cost compared to the geometry optimizations typically employed by other algorithms. As an additional benefit, sampling of configurations beyond optimized structures is known to enhance the potency^30^ of the training data by increasing the diversity in atomic environments.

The DFT data is used to fit the ANN that drives the GA. When the GA encounters new clusters inside the extrapolation regime of the ANN, these structures are reevaluated using DFT. If the error in the predicted energy exceeds the 2σ-range of the ANN model, the relevant clusters are added to the data set and the ANN is reparametrized. Following this procedure, new structures are added iteratively to the global data set during exploration of the various systems. This allows the network to benefit from structural information accumulated during earlier studies. As the size of the data set grows, so does the interpolation domain. The odds of reparametrization during the GA procedure therefore diminish, leading to efficient exploration of the configuration space.

Typical errors for the ANN generated in this work range around 1–2 meV/atom. These values are higher than those reported in earlier studies on molecular adsorption.^30^ The higher error originates from the smaller data sets relative to the degrees of freedom of the clusters. The reported accuracies are high enough for use in the GA, as they are found not to affect the identification of stable structures. Errors in the energies affect the determination of trial move selection, though the larger number of clusters that is generated by the ANN compensates for these minor deviations in transition probabilities by providing a more thorough exploration of the cluster configurations. In order to eliminate residual errors, a final DFT optimization is performed for the clusters in the low-energy metastable ensemble (LEME) before these structures are employed in subsequent analyses.

To close the loop (see Figure 2), the LEME ensemble is used to expand upon the existing transfer data set of cluster configurations which in turn can be used to spawn new cluster data sets. It is important to mention that the transfer data set itself does not store the chemical identity of the atoms, only their positions. To enable scaling of the interatomic bond lengths to those of the target elements, structures in the transfer set are scaled to a common reference which may be kept as meta-data. This procedure removes the need to track compositional information for individual samples while simultaneously streamlining sample processing workflows. Chemical identities are only added to the samples during the construction of the initial cluster data set, resulting in a versatile procedure for the exploration of nanoclusters of different metallic compositions.

## 3 Results and Discussion

The performance of the transferable data sets is investigated by considering supported PtSn and NiIn clusters. Reference structures from literature studies are used to initialize the data sets. Results are compared against a DFT-driven GA. Initial focus is placed on the study of alloy systems given the strong structural similarity that is expected for clusters with varying alloy ratios. Transferability of the data sets across different systems is tested by comparing data set performance for both PtSn and NiIn. Subsequent analysis is then provided for the extension of these data sets to purely metallic systems, as these showcase new structures with increasing crystallinity. The effect of the support geometry is investigated by considering nonflat surface terminations. Finally, the scaling of the method is analyzed by considering increasing metal cluster sizes.

### 3.1 PtSn Alloys

Initial application of the transfer data sets is shown for supported 8-atom PtSn nanoclusters. The Pt catalyst is primarily utilized for hydrogenation of organics and performs an important role in oil refining and petrochemistry.^31−33^ Sn-alloying has been shown experimentally to enhance the catalytic activity.^34,35^ Formation of the alloy phase is seen to coincide with a reduction in particle size, suggesting PtSn nanocluster formation. Small clusters of 8 atoms were therefore considered, providing a lower limit for probing Pt–Sn interactions. PtSn particles are generated with a Sn content ranging from 0 to 8 atoms, the latter corresponding to the pure Sn phase, in order to study the effect of the alloy composition on the cluster structure. Al_2_O_3_ is most commonly utilized as the support for this catalyst, though recent studies have shown enhanced activity for CeO_2_.^31,35^ Transferability of the data set was therefore utilized to investigate the role of the support in the cluster formation.

#### 3.1.1 PtSn/Al_2_O_3_

**Figure 3:**
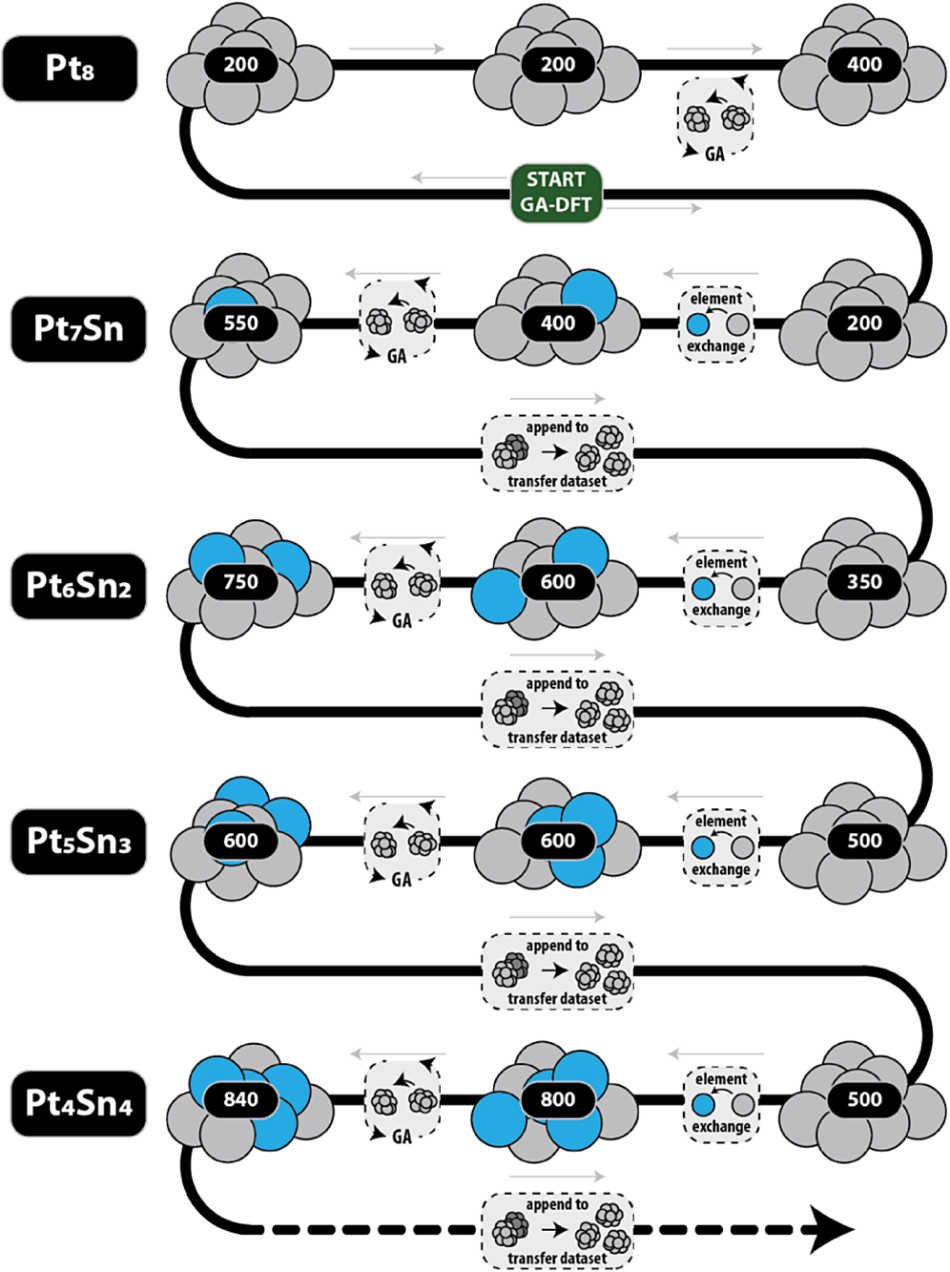
Transfer data set utility during PtSn/Al_2_O_3_ cluster-compositional exploration. Starting from an initial GA-DFT data set with 200 structures, ANN training sets are generated through chemical substitution. Subsequent ANN-driven GA explorations append new cluster geometries to the growing data set, benefiting downstream studies.

A DFT-driven GA was used to explore the low-energy metastable ensemble (LEME) of Pt_8_/Al_2_O_3_. This set contains the structures near the global energy minimum and provides an indication of the geometries that are accessible at elevated temperatures. 200 distinct structures, including the high-energy candidates, were generated during the GA cycles. This set of structures is used to initialize the transfer data set, as illustrated in Figure 3. Upon substitution of a single Sn atom into a Pt cluster, the structure of the cluster is not expected to change dramatically. A transfer data set based on Pt_8_ should then provide a reasonable sampling of the configurational space of the Pt_7_Sn clusters. The training data set for the Pt_7_Sn-ANN was generated through random substitution of the Pt atoms in the transfer data set. 400 structures were generated initially. Single-point calculations were performed for these structures and a network was fitted. (Details are available in the Supporting Information.)

The Pt_7_Sn-ANN was used to perform a GA search for the corresponding LEME structures. During the GA search, new structures were encountered possessing atomic environments not represented by the reference data set. While the majority of these structures ended up high in energy, they were still required to inform the GA. New structures were added to the transfer data set 7 times during the full GA search, adding 150 structures in total. The ANN was reparametrized each time to account for the new data.

For reference, a Pt_8_-ANN was created. The set of 200 structures in the transfer data set was found to be insufficient to yield an accurate parametrization. An additional 200 geometries were obtained from the geometry optimization trajectories to satisfy the desired performance. Addition of the intermediate structures had only a minor effect on the diversity of atomic environments. Their necessity was linked to the propensity of the ANN to overfit the small data set, leading to early convergence of the optimizer. Here, the small data set size provides an insufficient number of samples for the network to uniquely determine the remaining coefficients during the latter stages of the optimization. The benefits of additional intermediate structures in the data set was found to be minimal, suggesting that there is simply a minimum size threshold to fit the mapping described by the data set. Further improvements would require new structural information to be added to the data set. This may be seen for the Pt_7_Sn-ANN, where a notable reduction in network RMSE is observed between data sets of 400 and 550 structures, respectively. The larger data set requirements for the alloy clusters traces to the extended configurational space following their reduced symmetry.

**Figure 4:**
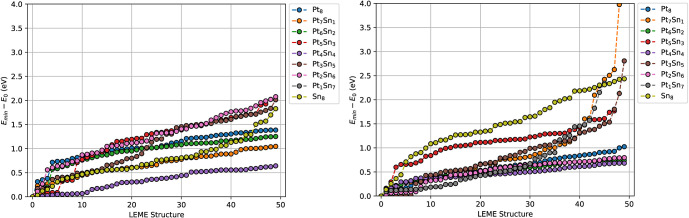
Low-energy structures obtained through ANN-driven GA searches for PtSn/Al_2_O_3_ (Left) and PtSn/CeO_2_ (Right).

The transfer data set was used to generate clusters with increasing Sn content. An overview is provided in Table 1. The final LEME energies are provided in Figure 4a. For Pt_6_Sn_2_, the shape of the LEME clusters changes substantially from those seen for Pt_8_ or Pt_7_Sn. Sn exhibits stronger interactions with the surface oxygen, resulting in formation of increasingly flat cluster geometries. An additional 150 structures were selected for refinement to accommodate extended sampling of this configurational regime. Initial data set sizes were increased as the size of the transfer data set grew. For Pt_5_Sn_3_, similar geometries were encountered as for Pt_6_Sn_2_. No additional samples were added to the data set. Observing the progression of the transfer data set size for the remaining clusters, the transfer set appears to have mostly converged, with samples spanning the full range of contact angles. 40 additional structures were added for Pt_4_Sn_4_, significantly less compared to the earlier extensions. The addition of the remaining 90 structures accounts for the strong Sn–O interactions of the high-Sn-content phases and serves to fill in any remaining gaps in the data.

**Table 1:** RMSE of Optimized ANN for PtSn/Al_2_O_3_ Nanoclusters given in meV/Atom^a^^b^^c^^d^^e^.

alloy	RMSE	*N*_*F*_	*N*_*I*_	*N*_*T*_
Pt_8_	1.104	400	200	200
Pt_7_Sn	1.058	550	400	200
Pt_6_Sn_2_	1.064	750	600	350
Pt_5_Sn_3_	1.170	600	600	500
Pt_4_Sn_4_	1.165	840	800	500
Pt_3_Sn_5_	1.060	800	800	540
Pt_2_Sn_6_	1.104	865	800	540
PtSn_7_	1.207	800	800	605
Sn_8_	1.211	330	300	605
Sn_8_	1.149	300	200	200
PtSn_7_	1.141	600	600	300
Pt_2_Sn_6_	1.082	700	600	300
Pt_3_Sn_5_	1.085	700	600	400
Pt_4_Sn_4_	1.164	820	800	500
Pt_5_Sn_3_	1.110	825	800	520
Pt_6_Sn_2_	1.223	815	800	545
Pt_7_Sn	1.150	825	800	560
Pt_8_	1.017	300	300	585

a A comparison is provided for transfer data sets initialized on Pt_8_ and Sn_8_.

b These data sets are refined following GA searches for each alloy.

c The size of the transfer data set at the start of each search is denoted by NT.

d The corresponding initial sample size for each system is denoted by NI.

e The final data set size is given by NF.

DFT-driven GA searches were performed for Pt_6_Sn_2_, Pt_4_Sn_4_, Pt_2_Sn_6_, and Sn_8_ to evaluate the performance of the ANN-driven GA. Several compositions were analyzed to compare the respective feature representations in the transfer data set. Both DFT and ANN methods found the same set of structures near the global energy minimum for each of the phases (to within numerically significant bounds). This showcases the capability of the ANN to replace DFT as the main driver for the GA searches. Comparing the sample distributions, the ANN has obtained a higher number of low-energy structures compared to DFT. Screening of permutations for Sn positions within the clusters is achieved to a greater extent by the ANN as a larger number of samples could be generated. This effect is particularly notable for the high-Pt-content phases, where many of these clusters lie close in energy.

Refinement of the transfer data set following the PtSn exploration yielded a total of 630 structures. A secondary transfer data set was constructed starting from a initial set of 200 Sn_8_ clusters (Table 1). Network performance and final data set sizes were found to be comparable. Initial expansion of the transfer data set is seen to occur predominantly following the random generation of new candidate geometries by the GA. Rapid convergence of the transfer data set size is noted, with subsequent GA searches for other phases being tied to differences in preferential geometry of the clusters. The stochastic sampling of the GA during the first several studies appears to gradually cover the cluster phase space, indicating little dependence on the order in which the phases are evaluated.

#### 3.1.2 PtSn/CeO_2_

The transfer data set generated for the Al_2_O_3_-supported clusters was used to initialize GA searches on the CeO_2_ support. Results are provided in Table 2 and the final LEME energies are shown in Figure 4b. The larger size of the starting transfer data set is seen to improve the initial parametrization of the networks. During application on Pt_8_, 110 new structures were added. These structures originate from the difference in lattice symmetry between the supports. The lattice oxygen is observed to have a strong templating effect on the interfacial metal atoms. This changes the internal coordination of the cluster atoms, which is not yet covered by the PtSn/Al_2_O_3_ data set. After this initial data set extension, GA searches for subsequent PtSn compositions see gradual incorporation of additional structures in line with the behavior for the Al_2_O_3_ system.

**Table 2:** RMSE of Optimized ANN for PtSn/CeO_2_ Nanoclusters Given in meV/Atom^a^^b^^c^

alloy	RMSE	*N*_*F*_	*N*_*I*_	*N*_*T*_
Pt_8_	1.121	510	400	800
Pt_7_Sn	1.125	800	800	910
Pt_6_Sn_2_	1.172	840	800	910
Pt_5_Sn_3_	1.171	840	800	950
Pt_4_Sn_4_	1.131	825	800	990
Pt_3_Sn_5_	1.296	810	800	1015
Pt_2_Sn_6_	1.145	805	800	1025
PtSn_7_	1.164	820	800	1050
Sn_8_	1.207	300	300	1050

a The transfer data set was initialized on PtSn/Al_2_O_3_ and subsequently refined during the alloy explorations.

b For each system, NT denotes the size of the transfer data set used to generate the initial sample set NI.

c The final data set size, after refinement, is given by NF.

By comparing the transfer data set growth between the supports, a clear bias toward the Al_2_O_3_ data can be observed. This follows as the initial content in the transfer data set was seen to provide the broadest configurational sampling. Consequently, a notable addition of samples was made when evaluating the CeO_2_-supported clusters. A reoptimization of the transfer data set samples was therefore conducted. This minimizes the overlap in features between samples and allows a reduction in the total data set size. A candidate data set is constructed by combining the GA samples collected for each composition for both supports. Redundant samples are filtered based on their CSR distance until a final transfer set of 900 structures was obtained. Results of GA searches for the optimized transfer set are provided in Table 3. No new samples were added during the explorations, indicating that the configurations represented by the optimized data set cover the range of structures encountered for both supports.

**Table 3:** RMSE of Optimized ANN for PtSn/Al_2_O_3_ and PtSn/CeO_2_ Nanoclusters Given in meV/Atom^a^^b^^c^

alloy	RMSE	*N*_*F*_	*N*_*I*_	*N*_*T*_
Pt_8_/Al_2_O_3_	1.061	400	400	900
Pt_7_Sn	1.213	900	900	900
Pt_6_Sn_2_	1.145	900	900	900
Pt_5_Sn_3_	1.078	900	900	900
Pt_4_Sn_4_	1.091	900	900	900
Pt_3_Sn_5_	1.207	900	900	900
Pt_2_Sn_6_	1.135	900	900	900
PtSn_7_	1.020	900	900	900
Sn_8_	1.163	300	300	900
Pt_8_/CeO_2_	1.166	400	400	900
Pt_7_Sn	1.066	900	900	900
Pt_6_Sn_2_	1.120	900	900	900
Pt_5_Sn_3_	1.108	900	900	900
Pt_4_Sn_4_	1.088	900	900	900
Pt_3_Sn_5_	1.225	900	900	900
Pt_2_Sn_6_	1.170	900	900	900
PtSn_7_	1.122	900	900	900
Sn_8_	1.105	300	300	900

a Transfer data sets were initialized on data from both supports.

b NT denotes the size of the transfer data set used to generate the initial sample set NI.

c The final data set size is given by NF.

#### 3.1.3 Comparison to DFT-GA

**Figure 5:**
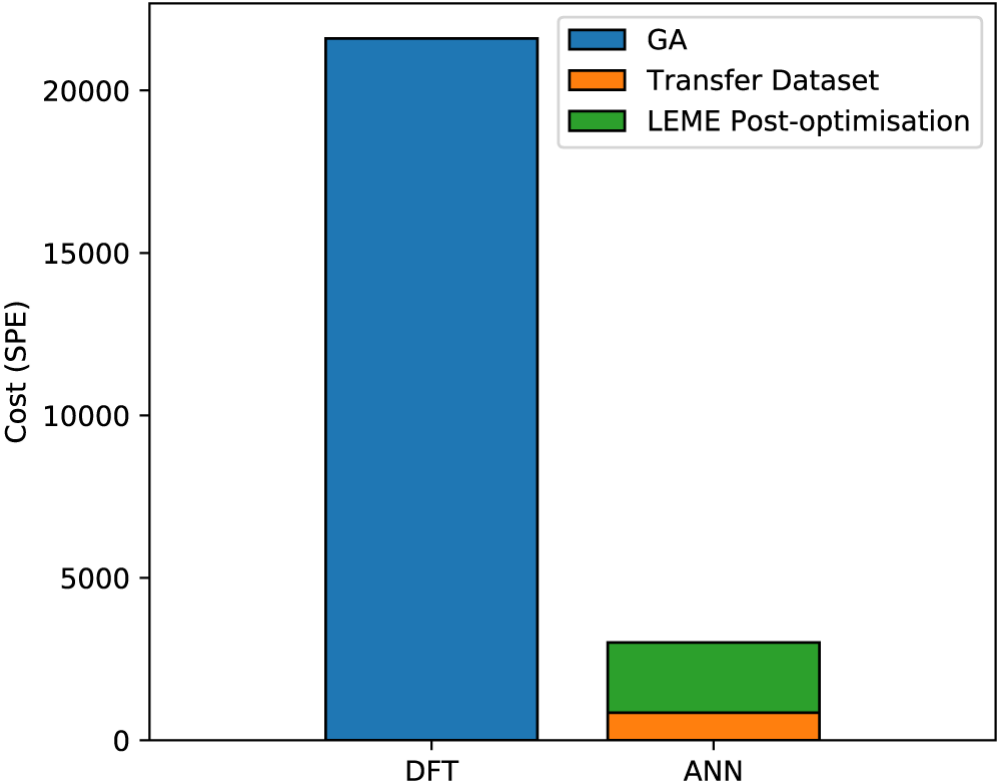
Average cost comparison between DFT- and ANN-driven GA searches for LEME structures of PtSn clusters.

To compare the cost of DFT and ANN for cluster exploration, single-point evaluations (SPE) are taken as reference, thereby accounting for variations in computation times between systems. One SPE is required per sample for the transfer data set. Exploration of the PtSn compositions required 800–900 samples per phase. DFT-driven GA searches performed for the PtSn clusters required around 200–400 samples to converge. Each of these samples requires a geometry optimization to be performed. Geometry optimizations in DFT constitute multiple single-point evaluations (SPE) in sequence. On average, 90 SPE were required per cluster. The cost of each SPE during geometry optimization is, on average, less than that of the ANN samples. The electron density obtained during each optimization step is used to preinitialize the next SPE, leading to faster convergence of the self-consistent field. The extent to which this affects the costs will be strongly dependent on the system. For PtSn, geometry optimization steps were found to be 20% cheaper on average. The total cost of GA-driven searches, assuming an average of 300 samples, is then equivalent to 21 600 SPE. The cost of the ANN construction was thus only 3–4% that of the full DFT-driven GA search (Figure 5). However, because the ANN leaves residual errors, resultant LEME structures will require a final DFT optimization prior to their use in follow-up studies. Assuming a LEME size of 30 structures, this increases the cost of the ANN method with an additional 10% relative to pure DFT. This estimate is by no means universal given the large variations in the number of GA structures, ANN data set sizes and geometry optimization costs. Regardless, as it may be assumed that many of these variations will cancel out, the cost reduction achieved by the ANN is expected to remain significant. The majority of the ANN costs are attributed to the postcorrection performed using DFT. These costs are static, as they scale only with the LEME size. The ANN have been shown to achieve this cost reduction without sacrificing the performance of the GA search. In fact, the lower costs of the ANN allow much larger sample sizes to be evaluated, increasing the odds of finding additional minima.

In addition to the reduction in CPU-time, front-loading of the computationally demanding tasks in the ANN-driven workflow helps streamline real-time planning. DFT-driven GA searches limit parallelization of the DFT calculations to the number of structures in the active cycle. The transfer data set meanwhile allows full batch-processing of all SPEs, enabling utilization of modern massively parallel computing infrastructures. The subsequent structure exploration is only interrupted when refinement of the data set is necessary. As the transfer data set improves, the number of interruptions is seen to decrease significantly. By reducing the cost of individual GA searches, compositional explorations may now be performed at the same cost as single-phase systems previously, increasing the accessible scope for this type of study.

### 3.2 NiIn Alloys

In_2_O_3_-supported Ni catalysts are currently under investigation for their performance in CO_2_ recycling to methanol.^29,36,37^ Bimetallic Ni–In catalysts were observed to yield higher selectivities.^36^ Increased catalytic activity of highly dispersed Ni/In_2_O_3_ suggests that Ni–In interactions may be responsible.^38^ Given that In atoms are relatively mobile compared to other oxide supports, NiIn nanoclusters have been proposed as the active phase. The alloy composition of the clusters is dependent on the interaction strength between metal and support. Similar to the previous analysis of PtSn nanoclusters, various compositions are evaluated to compare the behavior of the clusters.

To seed the ANN data set, 200 Ni_8_ structures were obtained from a previous study employing DFT-based GA.^29^ An ANN is constructed for Ni_4_In_4_ in order to gain insight into the Ni–In interaction. Through random substitution of In atoms, 600 candidate structures are generated for fitting the ANN. During subsequent GA cycles, 6 reinitializations were required, with an additional 200 structures being appended to the data set. These structures possess geometric features which differ significantly from those encountered for Ni_8_. GA explorations into the structure of the remaining compositions were performed in order of increasing In content (Table 4). During investigation of Ni_6_In_2_, additional refinements were performed upon addition of 110 new candidate structures. Remaining alloy compositions did not require reoptimization of the ANN. In the case of In_8_, formation of flat overlayers is observed as the binding strength between atoms in the cluster and the surface are nearly identical. These flat structures were not previously included in the data set. Only around 20 structures were required to fit this behavior due to the similar environment for all cluster atoms. Filtering of redundant structures from the completed transfer set yielded a definitive set of 480 structures. The size of this transfer set is similar to those encountered for single-support PtSn. The transfer methodology thus achieves comparable cost reduction relative to DFT as for the PtSn systems.

**Table 4:** RMSE of Optimized ANN for NiIn/In_2_O_3_ Nanoclusters Given in meV/Atom^a^^b^^c^.

alloy	RMSE	*N*_*F*_	*N*_*I*_	*N*_*T*_
Ni_8_	1.259	350	350	200
Ni_4_In_4_	1.343	800	600	200
Ni_7_In	1.262	800	800	400
Ni_6_In_2_	1.321	910	800	400
Ni_5_In_3_	1.371	800	800	510
Ni_3_In_5_	1.345	800	800	510
Ni_2_In_6_	1.340	800	800	510
NiIn_7_	1.205	800	800	510
In_8_	1.193	530	510	510

a The transfer data set was initialized on Ni_8_/In_2_O_3_ and subsequently refined during the alloy explorations.

b NT denotes the size of the transfer data set used to generate the initial sample set NI.

c The final data set size is given by NF.

#### 3.2.1 NiIn-PtSn Transfer

The goal of the transfer data set construction is to obtain a system-agnostic reference set for the cluster geometries. Two independent data sets have been constructed for PtSn and NiIn. Transferability of these data sets to the complementary system has been investigated (Table 5). In both cases, some refinement of the initial transfer data set is observed. Given that both Ni and Pt possess the same crystal structure, this refinement mostly accounts for the difference in support structure. The transfer of structures from chemically distinct systems aids in the initialization of the ANN and yields a reduction in computational cost that is comparable to the compositional analyses performed earlier. Refinement remains necessary to account for differences in the dominant configurational subdomains.

**Table 5:** RMSE of Optimized ANN for PtSn and NiIn Nanoclusters Given in meV/Atom^a^^b^^c^^d^

alloy	RMSE	*N*_*F*_	*N*_*I*_	*N*_*T*_
Pt_8_	1.141	410	400	480
Pt_7_Sn	1.148	816	800	490
Pt_6_Sn_2_	1.147	800	800	506
Pt_5_Sn_3_	1.191	810	800	506
Pt_4_Sn_4_	1.126	810	800	516
Pt_3_Sn_5_	1.304	800	800	526
Pt_2_Sn_6_	1.192	800	800	526
PtSn_7_	1.124	800	800	526
Sn_8_	1.203	402	400	526
Ni_8_	1.187	420	400	900
Ni_4_In_4_	1.258	940	920	920
Ni_7_In	1.281	940	940	940
Ni_6_In_2_	1.402	952	940	940
Ni_5_In_3_	1.306	952	952	952
Ni_3_In_5_	1.258	952	952	952
Ni_2_In_6_	1.304	952	952	952
NiIn_7_	1.173	956	952	952
In_8_	1.200	400	400	956

a The transfer data set for PtSn was initialized on NiIn data and vice versa.

b These data sets were iteratively refined following GA searches on each alloy.

c NT denotes the size of the transfer data set used to generate the initial sample set NI for each system.

d The final data set sizes are given by NF.

Merging of the PtSn and NiIn data sets, followed by filtering of redundant features, yielded a new reference set of 960 structures. 260 structures originated from NiIn. This reflects the relative distribution of PtSn:NiIn samples included in the data set. The large reduction in total reference set size was possible as ANN input is defined on a per-atom basis. Samples generated during the GA tend to be strongly correlated, resulting in sample variations originating from a subset of atoms. The CSR filter prioritizes highly uncorrelated samples, increasing the amount of relevant information included per structure. Application of the global reference set to PtSn and NiIn is shown in Table 6. A handful of refinement stages occurred during this process. Previously, merging of data sets for PtSn did not show the need for refinement. The occurrence of refinement despite the optimized data sets was found to simply be an artifact from the stochastic GA. The new samples that were added during refinement did not match any of the structures discarded during filtering, and were furthermore found to lie high in energy. This indicates that the data set optimization achieved size reduction without limiting information for the ANN.

**Table 6:** RMSE of Optimized ANN for PtSn and NiIn Nanoclusters given in meV/Atom^a^^b^^c^^d^

alloy	RMSE	*N*_*F*_	*N*_*I*_	*N*_*T*_
Pt_8_	1.099	410	400	960
Pt_7_Sn	1.119	970	970	970
Pt_6_Sn_2_	1.151	970	970	970
Pt_5_Sn_3_	1.129	974	970	970
Pt_4_Sn_4_	1.134	974	974	974
Pt_3_Sn_5_	1.300	974	974	974
Pt_2_Sn_6_	1.118	974	974	974
PtSn_7_	1.183	974	974	974
Sn_8_	1.188	400	400	974
Ni_8_	1.149	400	400	960
Ni_4_In_4_	1.142	980	960	960
Ni_7_In	1.253	982	980	980
Ni_6_In_2_	1.166	982	982	982
Ni_5_In_3_	1.343	982	982	982
Ni_3_In_5_	1.276	982	982	982
Ni_2_In_6_	1.323	982	982	982
NiIn_7_	1.186	982	982	982
In_8_	1.184	400	400	982

a The transfer data set contained both PtSn and NiIn data.

b Iterative data set refinement was conducted during GA searches on both PtSn and NiIn subsystems.

c NT denotes the size of the transfer data set used to generate the initial sample set NI for each alloy.

d The final data set sizes are given by NF.

#### 3.2.2 Accessible Structures

**Figure 6:**
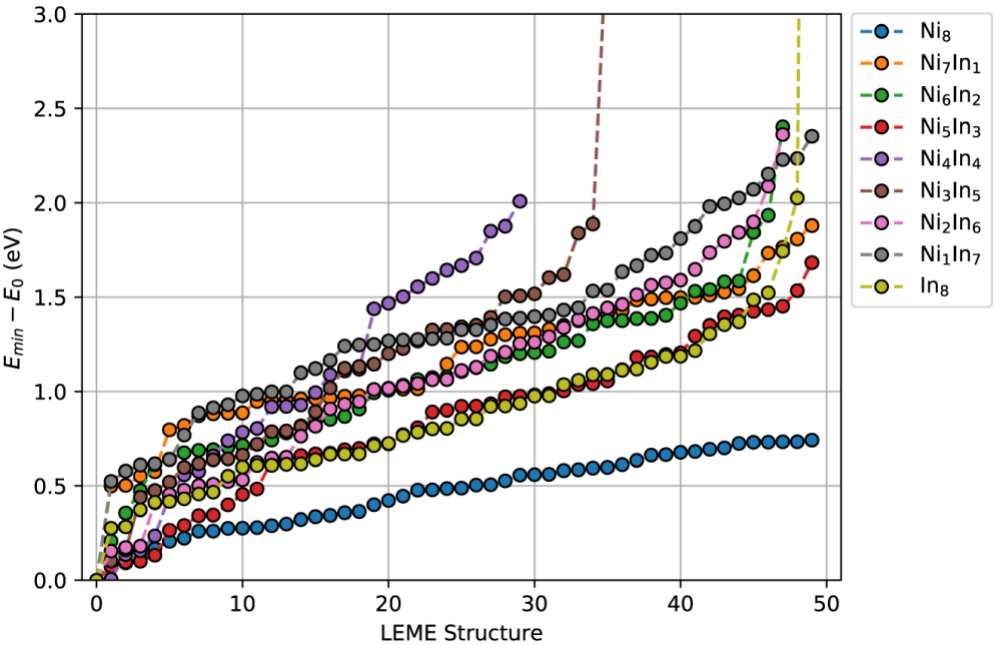
Low-energy structures obtained through ANN-driven GA searches for NiIn/In_2_O_3_.

The objective of the GA searches is to determine the set of structures that is accessible during operating conditions. While a single structure is identified as being the most stable, multiple structures are likely to coexist on the catalyst support at typical operating temperatures. In order to estimate the accessible structures, a Boltzmann population analysis may be performed. The canonical partition function is used to determine the likelihood for occupying each energy level at a given temperature, thereby accounting for thermal interconversion between structures. For all particles considered in this work, the LEME set is found to contain only 1–5 accessible structures per phase (Figures 4 and 6).

Relatively large energy level spacings appear to occur consistently in the distributions of the various particle systems. These energy ranges are not strictly empty, instead being occupied by vibrational distortions of the ground state structures. Within typical operating temperature ranges, only a handful of distinct structures will therefore be accessible. This degree of homogeneity contradicts experimental observations, however, as high polydispersity is typically observed. Two possible explanations may be given. First, this structural diversity may originate during synthesis or pretreatment of the catalyst. During these stages, high temperatures or reactive potentials may be experienced which make high-energy states accessible. However, low kinetic barriers to changes in the cluster structures invariably result in decay to lower-energy states. Therefore, a more likely scenario attributes the diversity to interaction with the adsorbates. High-energy clusters are less stable, resulting in the possibility of enhanced activation of reactants. Adsorption may stabilize these high-energy states, making them accessible during the reaction. The cluster-adsorbate system will then pose a different energy distribution compared to the vacuum state. Furthermore, binding to the adsorbate may result in structural changes in the cluster. This follows as the binding character differs for each of the vibrational energy levels. These structural changes may result in formation of new, stable geometries within the thermally accessible energy window. Existing networks can be extended by including coverage data to account for metal–adsorbate interactions. The influence of the adsorbates can then be investigated as part of the GA searches.

**Figure 7:**
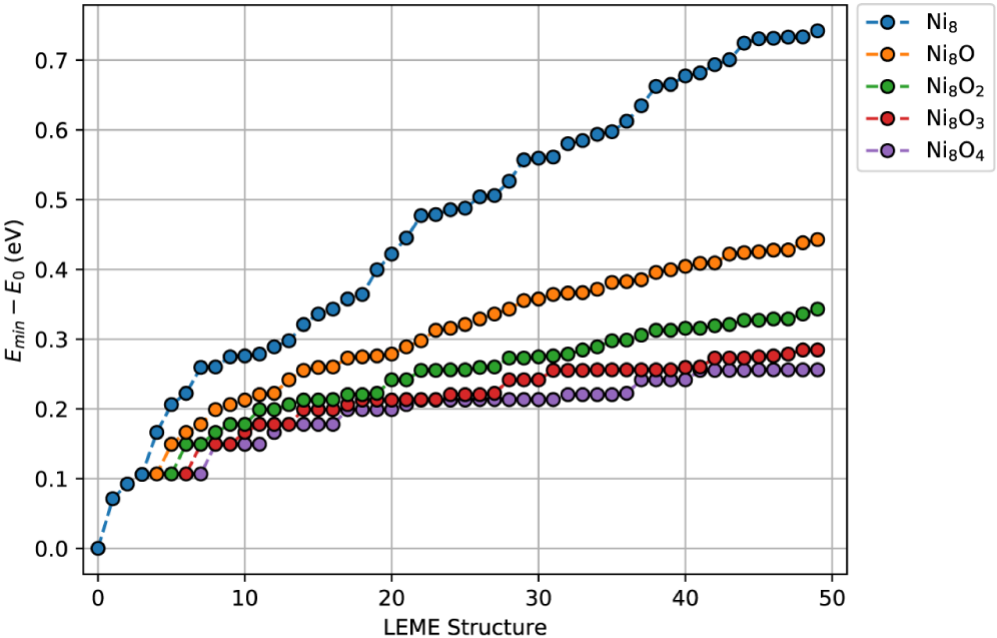
Low-energy structures obtained through ANN-driven GA searches for O/Ni_8_/In_2_O_3_.

An example of this reparametrization has been provided for oxygen spillover onto Ni_8_. The energy level density is seen to increase quite drastically due to the additional configurational freedom afforded by the oxygen atoms (see Figure 7). Various adsorption sites yield different binding energies, represented by distinct energy levels. These energy differences are comparable to the energy level spacing observed for the bare clusters, resulting in overlap in the bands associated with each structure. As the LEME set of Ni_8_ consists mostly of highly crystalline structures, similar geometries remain stable upon oxygen adsorption, with relatively minor distortions of the bare structures. This behavior may be different for other metals or alloys, where significant structural reorganization could occur. The presented workflow can readily account for such changes through extension of the transfer data set.

### 3.3 Effect of Support Structure

The supports considered thus far possess reasonably flat geometries. In turn, the flatness of the electron density isosurface yields similar cluster-metal interactions along the translational grid. The stable cluster geometries will therefore have a similar shape at most of the surface sites by virtue of this templating effect. In order to probe the impact of surface corrugation on the effectiveness of the transfer data sets, TiO_2_-supported clusters were evaluated. For this exploration, we consider a pure Co system and investigate the effect of various supports: Al_2_O_3_, CeO_2_, SiO_2_ and both rutile and anatase polymorphs of TiO_2_.

LEME structures of the supported clusters may be found in the Suppoting Information. Application of the transfer method for Co_8_ clusters did not require further refinement of the data set for any of the supports (Table 7). This reflects the extended sampling that has been performed prior. For Al_2_O_3_ and CeO_2_, LEME structures resemble those found for Pt and Ni. This similarity likely originates from the templating effect of the support. SiO_2_ has similar symmetry to CeO_2_. The larger interatomic spacings yield a mixture of clusters with various contact angles. These structures resemble those encountered for the alloys previously and are thus accurately covered by the transfer data set. For the TiO_2_ supports, larger initial data set sizes are required to account for the lower symmetry of these surfaces during translational sampling. Once the interaction between the support and the clusters has been fitted by the ANN, the subsequent set of clusters that was generated resembled those encountered for CeO_2_. Changes in the structure of the support were found to be minor, such that the parametrization was seen to hold throughout the GA cycles.

**Table 7:** RMSE of Optimized ANN for Co_8_ Nanoclusters given in meV/Atom^a^^b^^c^

support	RMSE	*N*_*F*_	*N*_*I*_	*N*_*T*_
Al_2_O_3_	1.116	960	960	960
CeO_2_	1.115	960	960	960
SiO_2_	1.171	960	960	960
Ant-TiO_2_	0.979	960	960	960
Rt-TiO_2_	1.008	960	960	960

a The transfer data set was initialized on PtSn and NiIn.

b NT denotes the size of the transfer data set used to generate the initial sample set NI for each system.

c The final data set size is given by NF.

#### 3.3.1 Data Set Transferability Between Metals

The transfer methodology has been shown to be successful for adsorption of Co clusters. Generalisation toward other metals is provided in Table 8. For CeO_2_, similar crystalline phases are observed for all metals due to a strong templating effect from the support. Hence, no refinement was expected for these systems. The presence of a diverse array of geometries in the transfer data set nevertheless remains important. After all, it cannot be concluded *a-priori* what the shape of the LEME clusters will be. This requires confirmation during the GA exploration. In addition, the stochastic nature of the GA has, at several instances, led to formation of high-energy structures, which the ANN must be able to accurately reject.

**Table 8:** RMSE of Optimized ANN for Mono-Metallic Nanoclusters Given in meV/Atom^a^^b^^c^

system	RMSE	*N*_*F*_	*N*_*I*_	*N*_*T*_
Rh_8_/CeO_2_	1.111	960	960	960
Ru_8_/CeO_2_	1.104	960	960	960
Ru_8_/Ant-TiO_2_	1.201	960	960	960
Ru_8_/Rt-TiO_2_	1.212	960	960	960
Ni_8_/Ant-TiO_2_	1.213	960	960	960
Ni_8_/Rt-TiO_2_	1.196	960	960	960

a The transfer data set was initialized on PtSn and NiIn.

b NT denotes the size of the transfer data set used to generate the initial sample set NI for each system.

c The final data set size is given by NF.

Ni_8_/TiO_2_ forms similar structures as Ni_8_/In_2_O_3_, resulting in few issues for the ANN. TiO_2_ primarily requires extended sampling of the translational variations in energy. LEME structures for both polymorphs appear to be structurally similar for all metals. Variations in energy distributions are mostly attributed to differences in the binding site geometry. Ru_8_/TiO_2_ forms simple cubic structures which were not seen during earlier GA searches. However, the ANN input vectors for these structures were covered by the CSR distribution of the transfer data set. Verification of the energy for these samples shows that they are accurately described by the ANN and thus did not require extension of the data set. The algorithm laid out for the transfer data set method thus appears quite capable in tackling a variety of metals, alloys and supports. New structures are reliably identified by analyzing the ANN input. Automated verification of uncertain samples allows for autonomous operation of the GA by including reparametrizations of the ANN. Given the broad range of systems for which the method has been shown to work, transfer data sets appear capable of dealing with all manner of nanocluster systems.

### 3.4 Scaling Toward Larger Particles

The previous sections have focused on the generation of 8-atom clusters to investigate the transferability of the data sets. Small nanoclusters are often employed in theoretical studies on account of their size to limit computational costs. With an increase in cluster size, the configurational space of the atomic configurations increases proportionally with the larger number of degrees-of-freedom. The transfer data set size is expected to increase accordingly. This scaling behavior was evaluated for the pure Co/SiO_2_ system by considering particles ranging from 6 to 20 atoms. For clusters below 6 atoms, direct sampling of the molecular degrees of freedom outperforms stochastic optimization.^30^

In order to initialize the transfer data set, up to now, we have utilized structures obtained from previous GA searches. Initial investigations required DFT-driven GA structures to seed the data set. With increasing cluster size, the cost of each DFT calculation increases following the increase in system size. In addition, the required number of sample evaluations increases due to the larger configurational space. The serial nature of genetic evolution results in extended wall-time for these simulations. While the total CPU-costs of this process are accessible on modern hardware, they are far from desirable. Two separate strategies have been developed to improve the transfer data set initialization.

1 An ANN is created using data on Co_8_. This provides an initial estimate of metal–support interactions. The GA may then be utilized in combinations with the ANN to perform an exploration of the larger clusters. This exploration does not provide an accurate estimation of the ensemble, but does yield an initial set of structures that may be utilized to seed the potential energy surface (PES) exploration through SPE.
2 Atoms may be appended to the existing Co_8_ clusters utilizing the same geometric potentials as used to determine the initial adsorption modes.

**Figure 8:**
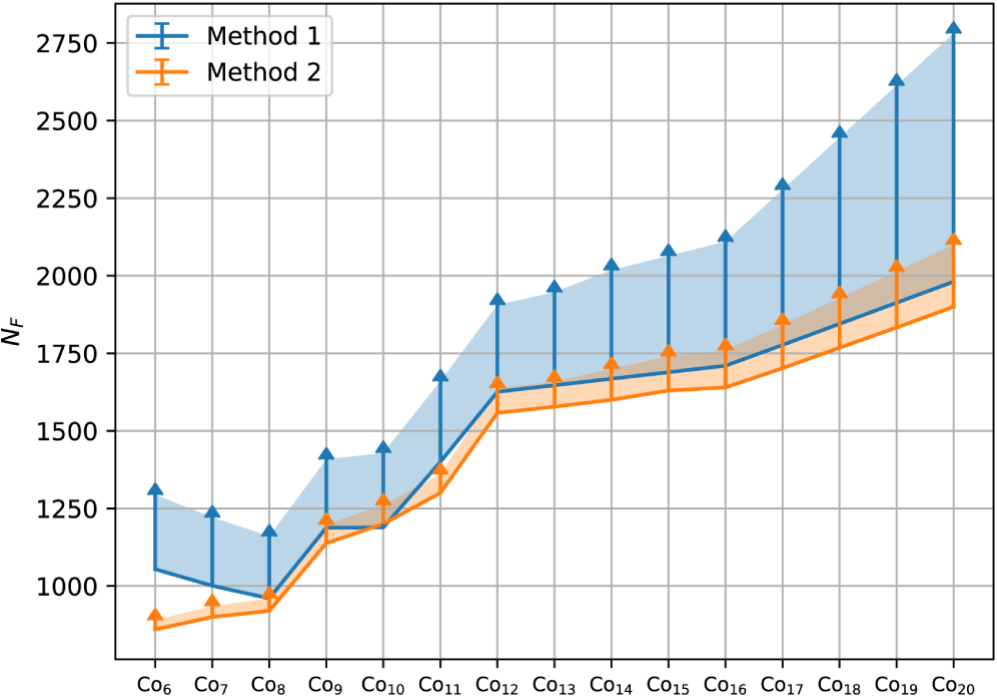
Final transfer data set sizes after ANN-driven GA searches for Co nanoclusters on SiO_2_. Two data set initialization methods were evaluated (as detailed in the discussion). For each cluster, 50 independent searches were performed. The spread in resultant sizes is highlighted.

A comparison of both strategies is provided in Figure 8. Multiple initializations have been performed to account for the stochastic nature of the GA. Variance in the ANN parametrization itself is accounted for by including a hyperparameter optimization as part of the network fitting. Both methods are seen to be capable of obtaining similar transfer data set sizes. However, the spread in values observed for Method 1 appears to be much larger. This variance in performance of Method 1 originates from the use of an ANN for the data set initialization. The distribution of basis function values differs quite a bit for clusters of different sizes. A range of structures will therefore fall in the extrapolation regime of the ANN. These structures may trap the GA in an unfavorable part of the PES. Poor initial guesses are eventually recovered by the GA as the sample density increases, however, this comes at the expense of larger data set sizes. By comparison, Method 2 appears to be more reliable for predicting cluster shapes as the geometric potential safeguards against unreasonable geometries. A minor spread in values is still observed, though this range is well accounted for by the GA stochasticity.

For Method 1, the increase in data set size for small clusters is reflective of poor guesses for these structures. The accuracy of the initial guesses diminishes as the difference in particle size increases. This follows as the structural features of these clusters are poorly represented in the training data for the ANN. Alternative data set construction methods may be devised to utilize data from clusters of various sizes, using, e.g., a process similar to the transfer methodology, where data from sequent cluster-size searches is utilized to improve the initial ANN parametrization. However, the diversity in structural features that is noted for the small clusters would cause extrapolation regimes to persist regardless. The consistency offered by Method 2 will therefore remain preferred.

The total data set size increases with the size of the cluster, reflective of the increase in size of the configurational space. The increase in cost with system size appears to be approximately linear with the number of atoms, where exponential scaling may be expected. This follows as the GA performs selective sampling of the stable structures. The exploration is restricted to a subset of the full cluster space, yielding more favorable scaling. Given the development of a crystalline core region for larger particles, this behavior is expected to persist, though further studies would need to be conducted to confirm this hypothesis. The spread in system size exhibits similar scaling, being proportional to the total data set size.

**Figure 9:**
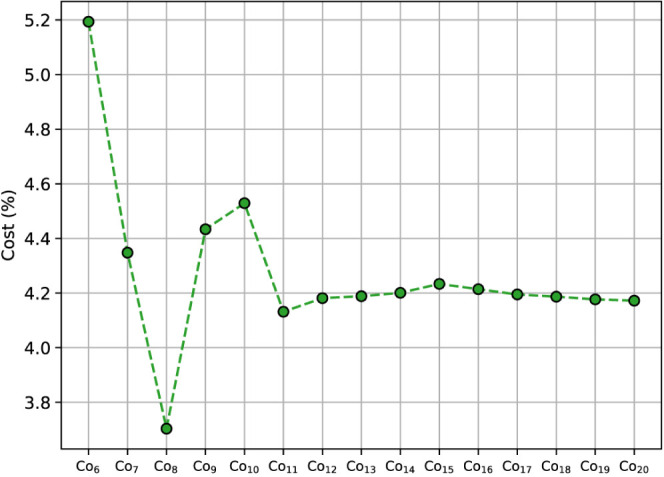
Relative cost of the transfer data set method for GA searches of Co nanoclusters on SiO_2_ compared to DFT-driven GA. ANN were initialized using structures obtained through adatom adsorption along a geometric potential. Median values are reported. The cost of DFT postoptimizations is not included.

A cost comparison between the DFT- and ANN-driven GA searches for the clusters is provided in Figure 9. Despite the linear increase in data set size, the relative cost reduction afforded by the ANN continues to hover around 4.2% for larger clusters. The number of structures evaluated by the GA increases with the system size, independently, for both DFT and ANN. The lower costs of the ANN are then awarded for circumventing geometry optimizations. Although the cost of the ANN is static once the network has been fitted, the larger configurational space of the particles results in frequent data set refinement, hindering further reduction in cost.

For small clusters, the efficiency of the ANN method is seen to diminish due to the smaller sample sizes for the DFT-driven GA. Meanwhile, the ANN still requires moderate data set sizes for accurate fitting. Structures in the intermediate regime show comparable costs. For Co_9_ and Co_10_, similar metal–support interactions are observed as for Co_8_. Parametrisation of the increased configurational space results in a proportional increase in transfer data set size. However, the templating effect of the support causes a large number of these structures to converge to the same minima. This limits the number of required cycles for the DFT-driven GA searches, lowering the cost relative to ANN. Formation of a more diverse LEME set is not seen to occur until larger clusters are formed.

The transfer data set methodology has been shown to reduce computational cost by ∼95% compared to DFT-driven GA searches. This efficiency is seen to persist for larger structures, enabling study of structure–activity relationships including metal–support interactions. Absolute costs of the LEME searches nevertheless continues to increase (as seen in Figure 8). The cost of the individual SPE also increases with system size, further taxing resources. When an ensemble of clusters with various sizes is of interest, performing independent GA searches for each cluster size can become inefficient. Improved initialization methods for disperse ensembles would be desirable to facilitate these studies. Initialisation of transfer data sets using structures of various sizes was not found to yield significant improvement in overall cost reduction as many refinement stages were required to account for the diversity in atomic environments of the nanoclusters. The efficiency of this approach may improve when clusters beyond 20 atoms are of interest, depending if the structural variety would be convergent. The development of targeted methods for direct sampling of the network input space would be of interest to improve the cost-efficiency as a means to counteract the SPE scaling.

#### 3.4.1 Performance for Unsupported Clusters

While the transfer data set approach has been illustrated for supported clusters, application to unsupported clusters is equally viable. Analysis of these systems is relevant for the investigation of homogenization mechanisms. Some studies have also utilized unsupported clusters as cheaper substitute models while avoiding simulation of the support material. For systems with weak metal–support interaction boasting larger particles, this approach allows for initial estimation of site distributions on the particles. A brief report of the performance of transfer data set construction for unsupported clusters is provided in the Supporting Information.

## 4 Conclusion

Transfer data sets have been developed for the automated exploration of nanocluster ensembles. A living database of cluster geometries is used to construct an ANN, which is employed in conjunction with genetic algorithms to search for stable structures. The ability to utilize single-point calculations to obtain reference DFT data, in contrast to geometry optimizations, allows for a reduction in computational cost of around 80–90%. Data generation lends itself to mass parallelization, enabling high-throughput studies. Orthogonalisation of the configurational subspaces reduces intersample correlation, improving initialization efficiency.

The performance of the transfer data sets has been compared to DFT-driven GA searches for a variety of metals, alloys and supports. Online refinement of the data set upon transfer between systems typically remains necessary due to the stochastic nature of the GA, yet is readily incorporated into the workflow. Gradual convergence of the data set features is observed as the transfer data set improves. Efficiency of the transfer method appears consistent across systems, suggesting general applicability of the presented approach. Scaling of the method toward larger particles was shown to preserve the reliability of the initialization procedure and the low cost of the transfer data set evaluation.

The transfer data set method was shown to be particularly suitable for alloy systems, as structural similarities are observed between phases. Application to disperse ensembles containing particles of varying sizes, where structural diversity is high, meanwhile exhibited little improvement over single-size searches. Additional multistage transfer methods may provide a solution to initialization of suitable transfer data sets for large-scale, disperse systems.

The cost-reduction allowed by the transfer methodology provides access to complex catalyst geometries compared to contemporary literature standards in kinetic or thermodynamic studies of catalytic systems. With numerous endeavors being focused on optimization of catalytic performance, the investigation of trends in catalyst composition, particle size or support effects is common. Construction of a local transfer repository allows leveraging the results of earlier subsystem studies to reduce downstream computational expense. These freed resources can be reallocated to investigate a broader range of model catalysts, or to increase per-sample cost by considering larger particles or complex catalyst–support interfaces.

## Data Availability

Data will be made available on request.

## Supporting Information Available

Details on ANN construction and parametrization, DFT and GA settings, and cluster geometries (PDF)
