# Supplementary material for: Transferable, Living Data Sets for Predicting Global Minimum Energy Nanocluster Geometries
Source: J Chem Theory Comput. 2024 Jul 24;20(15):6801–12. doi: 10.1021/acs.jctc.4c00572 (PMC11325533; doi:10.1021/acs.jctc.4c00572)
Supplement: Supplementary file 1 — ct4c00572_si_001.pdf [file ct4c00572_si_001.pdf]

Supporting Information: Transferable, Living Datasets for Predicting Global Minimum Energy Nanocluster Geometries

Bart Klumpers, Emiel Hensen, Ivo Filot*

* Laboratory of Inorganic Materials and Catalysis, Department of Chemical Engineering and Chemistry, Eindhoven University of Technology, 5600 MB, Eindhoven, The Netherlands, E-mail: i.a.w.filot@tue.nl

Contents

1 Computational Details **2**
1.1 ANN Construction 2
1.2 Genetic Algorithm 3

2 Network Performance **4**

3 LEME Structures **9**

4 DFT-ANN LEME Comparisons **23**

5 Unsupported Co Clusters **23**

1 Computational Details

1.1 ANN Construction

A basis set of radial (R), angular (A) and electronic (B) sampling functions is used to convert the coordinates of the structures in the dataset into feature vectors that are used as input for the ANN.

$$R_i = \sum_j g_c\left(r_{ij}\right) e^{-\eta r_{ij}^2} \tag{S1}$$

$$\begin{aligned} A_i = &\sum_j \sum_{k>j} g_c\left(r_{ij}\right) g_c\left(r_{ik}\right) e^{-\eta\left(r_{ij}^2 + r_{ik}^2\right)} \\ &\times \left[\lambda \frac{r_{ij} \cdot \vec{r_{ik}}}{r_{ij} r_{ik}} + (1-\lambda) \frac{\|\vec{r_{ij}} \times \vec{r_{ik}}\|}{r_{ij} r_{ik}}\right]^\zeta \end{aligned} \tag{S2}$$

$$B_i = \varepsilon_i + \sum_j \left(\varepsilon_i - \varepsilon_j\right) H\left(r_c - r_{ij}\right) \tag{S3}$$

Evaluated here for atom i. Interactions sum over all neighbours within a cut-off radius r_c. r_{ij} denotes the distance between atom i and neighbour j. ε denote scalar atomic parameters.

$$g_c\left(r\right) = \begin{cases} \frac{1}{2} + \frac{1}{2}\cos\left(\pi \frac{r}{r_c}\right) & r \leq r_c \\ 0 & r > r_c \end{cases} \tag{S4}$$

$$H\left(r\right) = \begin{cases} 0 & r < 0 \\ 1/2 & r = 0 \\ 1 & r > 0 \end{cases} \tag{S5}$$

The basis set for the ANN was optimised using an iterative CSR method.[1] A cut-off radius of 6.5 Å includes interactions with the third coordination shell for the metal atoms. The set of radial decay parameters η included 0, 0.025, 0.05, 0.15, 0.25, 0.5 and 1.0 Å^{-2}. The angular decay parameters ζ included 1, 1.5, 3, 6, 16 and 32. Two phase offsets $\lambda = 0, 1$ were included for the angular sampling. This yields 91 structural basis functions in total. The core charge, valence charge and magnetic moment were included as atomic parameters for the electronic basis set.

The ANN coefficients were optimised using the RMSprop optimiser coupled with a single-variable descent stage. The initial coefficients were obtained from the Glorot uniform initialiser.[2] Gradients were calculated using mini-batches of 50 samples. A training set containing 80% of samples was used for the conjugate gradient evaluations. 10% of samples was used as a validation set to evaluate convergence of the optimiser while the remaining 10% was used as a test set to determine subset biasing. These subsets were generated randomly from the available data. The system was said to be converged if the validation root-mean-square error (RMSE) did not improve over 300 iterations.

A hyperparameter screening was performed in order to determine the optimal network architecture, wherein it was chosen to use the same architecture for

the atomic networks for each unique element. A penalty function, as introduced previously was used to determine the optimum.

$$\varepsilon = \varepsilon_{Tr} + |\varepsilon_{Tr} - \varepsilon_V| + |\varepsilon_{Tr} - \varepsilon_{Te}| \tag{S6}$$

Where ε_{Tr} the error of the training set, ε_V the error of the validation set and ε_{Te} the error of the test set. This allows identification of the architecture corresponding to the lowest overall error (characterised by the training set) while accounting for subset biasing, overfitting and underfitting by considering the offset against the validation and test sets. The hyperbolic tangent was used as the non-linear function. (Sigmoids were included in the optimisation, but were consistently outperformed.) The number of layers was allowed to range from 1 to 5. The number of nodes was varied from 10 to 50 using increments of 5. For each configuration, 50 networks were generated to account for variance during the fitting.

ANN were used to perform the energy calculations for the GA. The GA was configured to generate 40 structures per cycle. The GA exploration was continued until at least 1000 clusters were generated. Once this target was achieved, the GA was set to converge when the LEME remained unchanged for 10 cycles. During the exploration, candidate clusters are analysed to identify extrapolation domains. If, during the GA cycles, structures were encountered that differ significantly from the dataset, the energy of these samples was verified using DFT. Structures were compared based on their CSR distance:

$$D_a = \min_b \sum_j^N \left| \frac{G_j^a - G_j^b}{N \cdot G_j^b} \right| \tag{S7}$$

G denotes the complete ANN input vector, a denotes the candidate cluster and b indexes the dataset samples. Permutational variance of atoms within the clusters was accounted for by concatenating like species in order of their vector norm. The CSR metric is used to identify the sample with the closest-matching fingerprint in the known dataset. Structures with a minimum distance exceeding 20% were verified using DFT. If the sample error exceeded the 2σ-range of the ANN, it was added to the dataset and the ANN was re-parametrised. The process continued until the structure exploration had converged.

1.2 Genetic Algorithm

A GA search was employed in order to obtain the set of structures near the energy minimum. To initialise the GA, initial candidate structures are obtained from the LEME of the transfer dataset. These clusters are adsorbed onto the selected support by utilising a geometric potential.[1] The GA will subsequently perform modifications to the candidate clusters while searching for the global energy minimum. First, clusters in the active cycle are subject to geometry optimisation. This converges the cluster to a local minimum. Convergence of the GA is then evaluated. The structure of the candidates is compared to the GA population. Clusters are considered to belong to the same minimum if their energy differs by less than 0.1 eV and the CSR distance between them is less than 5%. Overlapping clusters are removed from the population. New candidate structures are generated by merging features from past candidates. Two parent

clusters are selected by sampling an exponential distribution around the global minimum-energy candidate:

$$U_i = \exp\left[-2\left(\frac{E_i - E_{min}}{E_{max} - E_{min}}\right)^2\right] \tag{S8}$$

The selected clusters are bisected by defining a dividing plane through each structure. This is done by randomly generating a surface normal through the geometric centre. One half from each parent is then used to generate a new candidate. Re-connecting these halves occurs along an inter-planar geometric potential to avoid high-strain configurations. Additional modifications to the candidate structures are performed to increase the sample diversity. 4 operations have been defined.

1. The cluster is translated along the support surface.
2. The cluster is rotated relative to the support surface.
3. A vibrational distortion is applied to the structure following the approach detailed in earlier work. [1]
4. Positions of the alloy constituents are shuffled. This option is disabled for mono-metallic clusters.

Each of these operations is assigned a 10% occurrence rate. The remaining 60% of candidates is left intact.

2 Network Performance

The optimised network architectures for each system are provided in Tables S1-S12.

Table S1: Optimised architectures for ANN of $PtSn/Al_2O_3$. Fitting was performed using a transfer dataset initialised on Pt_8. RMSE and biases are reported in meV/atom. N denotes the number of sequential transforms in the network. W denotes the output dimension of the linear transforms.

Alloy	N	W	ε_T	b_T	ε_{Tr}	b_{Tr}	ε_V	b_V	ε_{Te}	b_{Te}
Pt_8	2	20	1.104	0.000	1.008	0.000	1.475	0.002	1.501	-0.002
Pt_7Sn_1	2	20	1.058	0.000	0.996	0.000	1.379	0.000	1.235	-0.001
Pt_6Sn_2	2	20	1.064	0.000	0.965	0.000	1.355	-0.001	1.565	0.001
Pt_5Sn_3	2	20	1.170	0.000	1.060	0.000	1.576	0.000	1.641	0.000
Pt_4Sn_4	2	20	1.165	0.000	1.077	0.000	1.568	0.002	1.465	-0.001
Pt_3Sn_5	2	20	1.060	0.000	0.921	0.000	1.556	0.002	1.677	-0.001
Pt_2Sn_6	2	20	1.104	0.000	1.035	0.000	1.422	-0.001	1.342	0.001
Pt_1Sn_7	2	20	1.207	0.000	1.121	0.000	1.477	0.001	1.624	-0.001
Sn_8	2	20	1.211	0.000	1.106	0.000	1.657	0.001	1.604	0.003

Table S2: Optimised architectures for ANN of PtSn/Al_2O_3. Fitting was performed using a transfer dataset initialised on Sn_8. RMSE and biases are reported in meV/atom. N denotes the number of sequential transforms in the network. W denotes the output dimension of the linear transforms.

Alloy	N	W	ε	b	ε_{Tr}	b_{Tr}	ε_V	b_V	ε_{Te}	b_{Te}
Pt_8	2	20	1.149	0.000	1.040	0.000	1.628	-0.002	1.541	-0.002
Pt_7Sn_1	2	20	1.141	0.000	1.035	0.000	1.540	0.003	1.592	-0.001
Pt_6Sn_2	2	20	1.082	0.000	0.969	0.000	1.626	0.001	1.439	-0.002
Pt_5Sn_3	2	20	1.085	0.000	0.974	0.000	1.544	0.002	1.513	0.001
Pt_4Sn_4	2	20	1.164	0.000	0.996	0.000	1.762	0.003	1.912	-0.001
Pt_3Sn_5	2	20	1.110	0.000	1.031	0.000	1.378	0.002	1.473	0.000
Pt_2Sn_6	2	20	1.223	0.000	1.098	0.000	1.736	0.002	1.708	0.000
Pt_1Sn_7	2	20	1.150	0.000	1.048	0.000	1.518	-0.001	1.599	0.000
Sn_8	2	20	1.017	0.000	0.942	0.000	1.297	0.001	1.340	0.001

Table S3: Optimised architectures for ANN of PtSn/CeO_2. Fitting was performed using a transfer dataset initialised on PtSn/Al_2O_3. RMSE and biases are reported in meV/atom. N denotes the number of sequential transforms in the network. W denotes the output dimension of the linear transforms.

Alloy	N	W	ε	b	ε_{Tr}	b_{Tr}	ε_V	b_V	ε_{Te}	b_{Te}
Pt_8	2	20	1.121	0.000	0.977	0.000	1.805	0.001	1.586	-0.002
Pt_7Sn_1	2	20	1.125	0.000	1.025	0.000	1.593	0.000	1.454	0.001
Pt_6Sn_2	2	20	1.172	0.000	1.092	0.000	1.434	0.002	1.548	0.001
Pt_5Sn_3	2	20	1.171	0.000	1.003	0.000	1.929	0.001	1.755	-0.001
Pt_4Sn_4	2	20	1.131	0.000	1.003	0.000	1.554	0.000	1.734	0.001
Pt_3Sn_5	2	20	1.296	0.000	1.194	0.000	1.691	0.001	1.719	0.001
Pt_2Sn_6	2	20	1.145	0.000	1.014	0.000	1.556	-0.001	1.781	-0.001
Pt_1Sn_7	2	20	1.164	0.000	0.997	0.000	1.910	0.000	1.752	0.002
Sn_8	2	20	1.207	0.000	1.041	0.000	1.886	-0.001	1.858	-0.001

Table S4: Optimised architectures for ANN of PtSn/Al_2O_3. Fitting was performed using a transfer dataset initialised on PtSn/Al_2O_3 and PtSn/CeO_2. RMSE and biases are reported in meV/atom. N denotes the number of sequential transforms in the network. W denotes the output dimension of the linear transforms.

Alloy	N	W	ε	b	ε_{Tr}	b_{Tr}	ε_V	b_V	ε_{Te}	b_{Te}
Pt_8	2	20	1.061	0.000	0.927	0.000	1.535	0.000	1.655	-0.001
Pt_7Sn_1	2	20	1.213	0.000	1.064	0.000	1.750	0.002	1.871	0.002
Pt_6Sn_2	2	20	1.145	0.000	1.066	0.000	1.546	0.000	1.372	0.003
Pt_5Sn_3	2	20	1.078	0.000	0.931	0.000	1.652	0.002	1.678	0.001
Pt_4Sn_4	2	20	1.091	0.000	0.949	0.000	1.646	0.003	1.670	0.001
Pt_3Sn_5	2	20	1.207	0.000	1.111	0.000	1.613	-0.001	1.565	0.000
Pt_2Sn_6	2	20	1.135	0.000	1.058	0.000	1.427	-0.001	1.462	0.003
Pt_1Sn_7	2	20	1.020	0.000	0.938	0.000	1.305	-0.002	1.392	0.000
Sn_8	2	20	1.163	0.000	1.071	0.000	1.590	0.000	1.470	0.002

Table S5: Optimised architectures for ANN of $PtSn/CeO_2$. Fitting was performed using a transfer dataset initialised on $PtSn/Al_2O_3$ and $PtSn/CeO_2$. RMSE and biases are reported in meV/atom. N denotes the number of sequential transforms in the network. W denotes the output dimension of the linear transforms.

Alloy	N	W	ε	b	ε_{Tr}	b_{Tr}	ε_V	b_V	ε_{Te}	b_{Te}
Pt_8	2	20	1.166	0.000	1.056	0.000	1.701	0.000	1.508	0.000
Pt_7Sn_1	2	20	1.066	0.000	0.965	0.000	1.468	0.000	1.474	0.001
Pt_6Sn_2	2	20	1.120	0.000	0.960	0.000	1.740	0.002	1.777	-0.001
Pt_5Sn_3	2	20	1.108	0.000	0.980	0.000	1.646	0.000	1.592	-0.001
Pt_4Sn_4	2	20	1.088	0.000	1.005	0.000	1.320	-0.001	1.522	0.002
Pt_3Sn_5	2	20	1.225	0.000	1.099	0.000	1.795	0.000	1.663	0.001
Pt_2Sn_6	2	20	1.170	0.000	1.052	0.000	1.705	0.002	1.579	0.002
Pt_1Sn_7	2	20	1.122	0.000	1.045	0.000	1.427	0.002	1.431	0.000
Sn_8	2	20	1.105	0.000	1.028	0.000	1.374	-0.002	1.454	-0.001

Table S6: Optimised architectures for ANN of $NiIn/In_2O_3$. Fitting was performed using a transfer dataset initialised on Ni_8/In_2O_3. RMSE and biases are reported in meV/atom. N denotes the number of sequential transforms in the network. W denotes the output dimension of the linear transforms.

Alloy	N	W	ε	b	ε_{Tr}	b_{Tr}	ε_V	b_V	ε_{Te}	b_{Te}
Ni_8	2	15	1.259	0.000	1.113	0.000	1.827	0.002	1.859	0.002
Ni_7In_1	2	20	1.343	0.000	1.159	0.000	2.122	0.001	2.032	0.003
Ni_6In_2	2	20	1.262	0.000	1.084	0.000	2.107	0.003	1.841	0.002
Ni_5In_3	2	20	1.321	0.000	1.234	0.000	1.656	0.001	1.680	-0.001
Ni_4In_4	2	20	1.371	0.000	1.173	0.000	2.315	0.001	2.014	-0.001
Ni_3In_5	2	20	1.345	0.000	1.183	0.000	1.918	-0.001	2.071	0.001
Ni_2In_6	2	20	1.340	0.000	1.189	0.000	1.943	-0.002	1.942	-0.001
Ni_1In_7	2	20	1.205	0.000	1.064	0.000	1.839	0.003	1.699	-0.001
In_8	2	20	1.193	0.000	1.098	0.000	1.584	0.002	1.559	0.002

Table S7: Optimised architectures for ANN of PtSn nanoclusters. Fitting was performed using a transfer dataset initialised on $NiIn/In_2O_3$. RMSE and biases are reported in meV/atom. N denotes the number of sequential transforms in the network. W denotes the output dimension of the linear transforms.

Alloy	N	W	ε	b	ε_{Tr}	b_{Tr}	ε_V	b_V	ε_{Te}	b_{Te}
Pt_8	2	20	1.141	0.000	1.018	0.000	1.633	-0.002	1.630	0.001
Pt_7Sn_1	2	20	1.148	0.000	1.048	0.000	1.607	-0.001	1.491	0.001
Pt_6Sn_2	2	20	1.147	0.000	1.045	0.000	1.585	0.002	1.527	0.002
Pt_5Sn_3	2	20	1.191	0.000	1.117	0.000	1.556	0.000	1.414	0.002
Pt_4Sn_4	2	20	1.126	0.000	1.000	0.000	1.528	0.002	1.732	0.001
Pt_3Sn_5	2	20	1.304	0.000	1.122	0.000	2.054	-0.001	2.008	0.001
Pt_2Sn_6	2	20	1.192	0.000	1.091	0.000	1.671	0.000	1.517	-0.001
Pt_1Sn_7	2	20	1.124	0.000	1.012	0.000	1.608	0.000	1.533	-0.002
Sn_8	2	20	1.203	0.000	1.117	0.000	1.431	-0.001	1.666	0.001

Table S8: Optimised architectures for ANN of NiIn/In_2O_3. Fitting was performed using a transfer dataset initialised on PtSn data. RMSE and biases are reported in meV/atom. N denotes the number of sequential transforms in the network. W denotes the output dimension of the linear transforms.

Alloy	N	W	ε	b	ε_{Tr}	b_{Tr}	ε_V	b_V	ε_{Te}	b_{Te}
Ni_8	2	15	1.187	0.000	1.025	0.000	1.956	0.000	1.717	0.002
Ni_7In_1	2	20	1.258	0.000	1.136	0.000	1.837	-0.001	1.655	0.002
Ni_6In_2	2	20	1.281	0.000	1.104	0.000	2.033	0.001	1.944	0.001
Ni_5In_3	2	20	1.402	0.000	1.301	0.000	1.789	0.000	1.824	-0.001
Ni_4In_4	2	20	1.306	0.000	1.167	0.000	1.894	0.000	1.829	0.002
Ni_3In_5	2	20	1.258	0.000	1.150	0.000	1.780	0.002	1.599	0.000
Ni_2In_6	2	20	1.304	0.000	1.171	0.000	1.949	0.002	1.726	0.001
Ni_1In_7	2	20	1.173	0.000	1.085	0.000	1.569	0.002	1.483	0.000
In_8	2	20	1.200	0.000	1.084	0.000	1.706	0.001	1.626	0.001

Table S9: Optimised architectures for ANN of PtSn nanoclusters. Fitting was performed using a transfer dataset initialised on PtSn and NiIn data. RMSE and biases are reported in meV/atom. N denotes the number of sequential transforms in the network. W denotes the output dimension of the linear transforms.

Alloy	N	W	ε	b	ε_{Tr}	b_{Tr}	ε_V	b_V	ε_{Te}	b_{Te}
Pt_8	2	20	1.099	0.000	1.025	0.000	1.394	-0.002	1.394	0.003
Pt_7Sn_1	2	20	1.119	0.000	0.996	0.000	1.622	-0.002	1.599	-0.002
Pt_6Sn_2	2	20	1.151	0.000	1.048	0.000	1.596	-0.002	1.529	0.000
Pt_5Sn_3	2	20	1.129	0.000	1.065	0.000	1.441	0.002	1.331	0.000
Pt_4Sn_4	2	20	1.134	0.000	0.980	0.000	1.703	0.000	1.795	0.003
Pt_3Sn_5	2	20	1.300	0.000	1.155	0.000	1.908	0.003	1.850	-0.002
Pt_2Sn_6	2	20	1.118	0.000	1.055	0.000	1.302	0.000	1.435	0.002
Pt_1Sn_7	2	20	1.183	0.000	1.082	0.000	1.629	-0.001	1.543	0.001
Sn_8	2	20	1.188	0.000	1.105	0.000	1.627	0.001	1.411	-0.002

Table S10: Optimised architectures for ANN of NiIn/In_2O_3. Fitting was performed using a transfer dataset initialised on PtSn and NiIn data. RMSE and biases are reported in meV/atom. N denotes the number of sequential transforms in the network. W denotes the output dimension of the linear transforms.

Alloy	N	W	ε	b	ε_{Tr}	b_{Tr}	ε_V	b_V	ε_{Te}	b_{Te}
Ni_8	2	15	1.149	0.000	1.077	0.000	1.394	-0.001	1.478	0.003
Ni_7In_1	2	20	1.142	0.000	0.980	0.000	1.769	0.001	1.807	0.003
Ni_6In_2	2	20	1.253	0.000	1.134	0.000	1.765	-0.002	1.696	0.001
Ni_5In_3	2	20	1.166	0.000	1.058	0.000	1.543	0.003	1.656	0.001
Ni_4In_4	2	20	1.343	0.000	1.237	0.000	1.695	0.001	1.843	-0.001
Ni_3In_5	2	20	1.276	0.000	1.149	0.000	1.643	0.000	1.925	-0.002
Ni_2In_6	2	20	1.323	0.000	1.198	0.000	1.857	0.003	1.792	0.001
Ni_1In_7	2	20	1.186	0.000	1.120	0.000	1.394	-0.001	1.506	-0.001
In_8	2	20	1.184	0.000	1.116	0.000	1.379	-0.002	1.532	0.000

Table S11: Optimised architectures for ANN of Co_8. Fitting was performed using a transfer dataset initialised on PtSn and NiIn data. RMSE and biases are reported in meV/atom. N denotes the number of sequential transforms in the network. W denotes the output dimension of the linear transforms.

Support	N	W	ε	b	ε_{Tr}	b_{Tr}	ε_V	b_V	ε_{Te}	b_{Te}
Al_2O_3	2	15	1.116	0.000	1.017	0.000	1.534	0.001	1.486	0.000
CeO_2	2	15	1.115	0.000	1.040	0.000	1.350	-0.001	1.477	0.001
SiO_2	2	15	1.171	0.000	1.035	0.000	1.753	0.001	1.677	-0.002
Ant-TiO_2	2	20	0.976	0.000	0.891	0.000	1.330	0.001	1.300	-0.001
Rt-TiO_2	2	20	1.008	0.000	0.888	0.000	1.608	-0.001	1.365	0.002

Table S12: Optimised architectures for ANN of mono-metallic nanoclusters. Fitting was performed using a transfer dataset initialised on PtSn and NiIn data. RMSE and biases are reported in meV/atom. N denotes the number of sequential transforms in the network. W denotes the output dimension of the linear transforms.

System	N	W	ε	b	ε_{Tr}	b_{Tr}	ε_V	b_V	ε_{Te}	b_{Te}
Rh_8/CeO_2	2	15	1.111	0.000	1.036	0.000	1.352	0.001	1.473	0.001
Ru_8/CeO_2	2	15	1.104	0.001	1.010	0.000	1.572	0.003	1.386	0.003
Ru_8/Ant-TiO_2	2	20	1.201	0.000	1.115	0.000	1.521	0.002	1.568	-0.001
Ru_8/Rt-TiO_2	2	20	1.212	0.000	1.063	0.000	1.724	0.001	1.892	0.003
Ni_8/Ant-TiO_2	2	20	1.213	0.000	1.125	0.000	1.462	-0.002	1.665	0.002
Ni_8/Rt-TiO_2	2	20	1.196	0.000	1.115	0.000	1.567	-0.001	1.477	0.000

3 LEME Structures

LEME trajectories for mono-metallic nanoclusters are given in Figures S1-S3. Reference DFT-driven GA searches for supported PtSn nanoclusters are given in Figure S4. Geometries for the low-energy structures of the various nanocluster systems are given in Figures S5-S42.

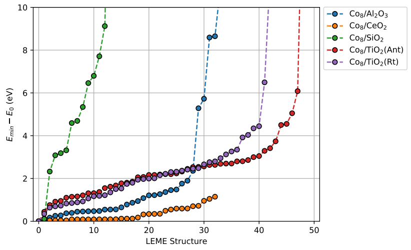

Figure S1: Low-energy structures obtained through ANN-driven GA searches for supported Co_8.

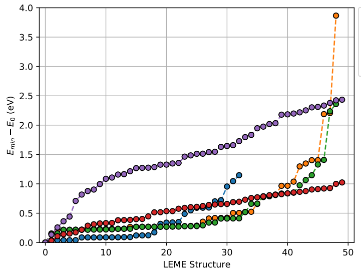

Figure S2: Low-energy structures obtained through ANN-driven GA searches for CeO_2-supported clusters.

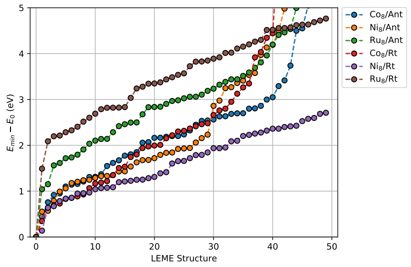

Figure S3: Low-energy structures obtained through ANN-driven GA searches for TiO_2-supported clusters.

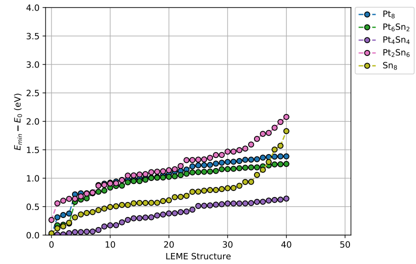

Figure S4: Low-energy structures obtained through DFT-driven GA searches for PtSn/Al_2O_3.

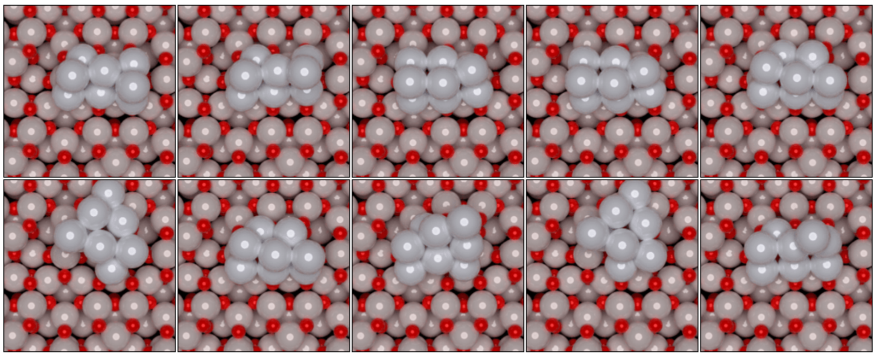

Figure S5: Geometries of the 10 lowest-energy clusters obtained through ANN-driven GA searches for Pt_8/Al_2O_3.

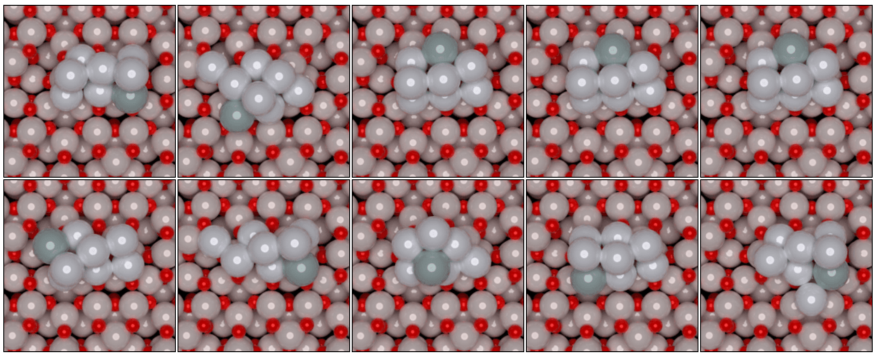

Figure S6: Geometries of the 10 lowest-energy clusters obtained through ANN-driven GA searches for Pt_7Sn_1/Al_2O_3.

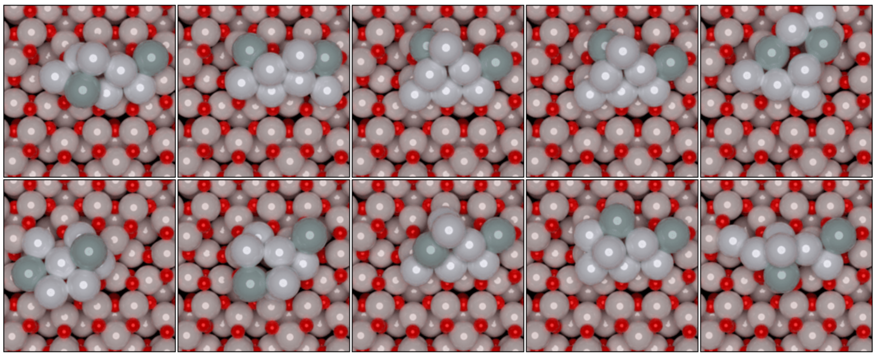

Figure S7: Geometries of the 10 lowest-energy clusters obtained through ANN-driven GA searches for Pt_6Sn_2/Al_2O_3.

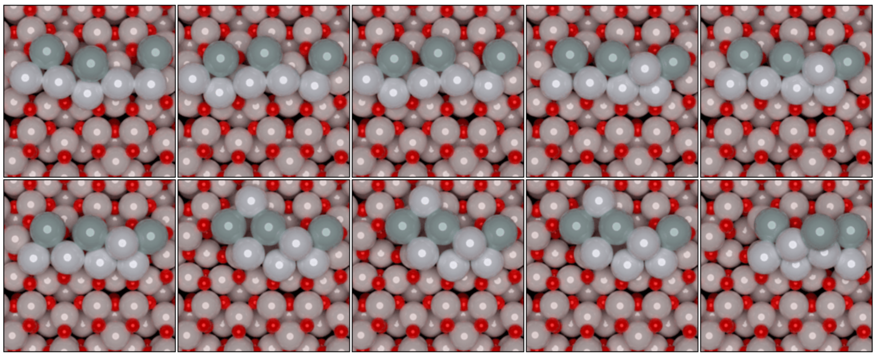

Figure S8: Geometries of the 10 lowest-energy clusters obtained through ANN-driven GA searches for Pt_5Sn_3/Al_2O_3.

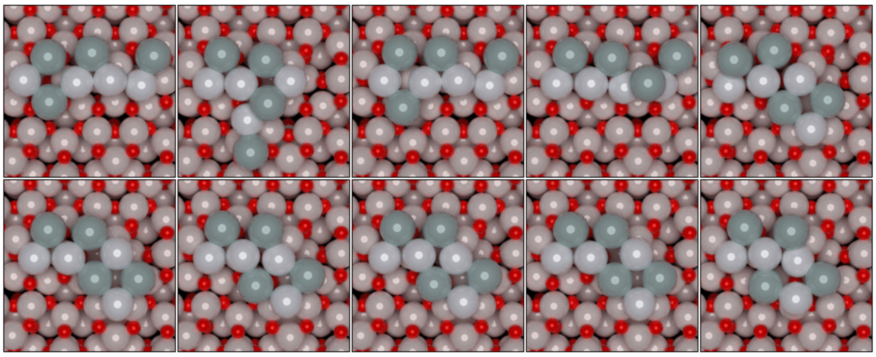

Figure S9: Geometries of the 10 lowest-energy clusters obtained through ANN-driven GA searches for Pt_4Sn_4/Al_2O_3.

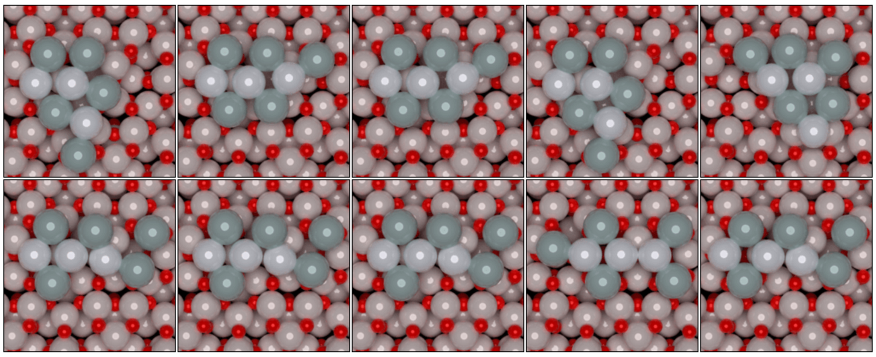

Figure S10: Geometries of the 10 lowest-energy clusters obtained through ANN-driven GA searches for Pt_3Sn_5/Al_2O_3.

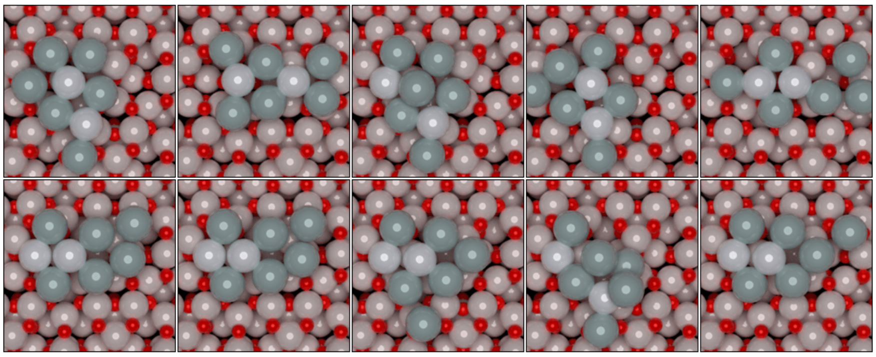

Figure S11: Geometries of the 10 lowest-energy clusters obtained through ANN-driven GA searches for Pt_2Sn_6/Al_2O_3.

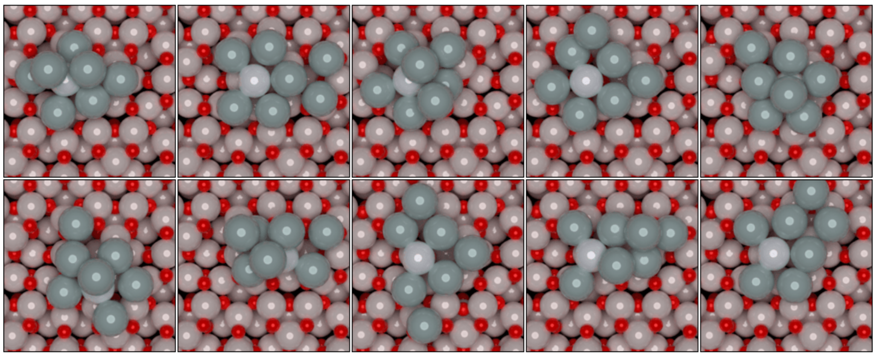

Figure S12: Geometries of the 10 lowest-energy clusters obtained through ANN-driven GA searches for Pt_1Sn_7/Al_2O_3.

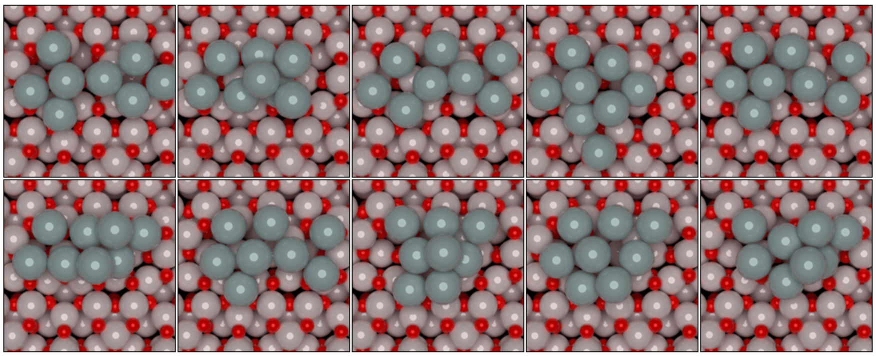

Figure S13: Geometries of the 10 lowest-energy clusters obtained through ANN-driven GA searches for Sn_8/Al_2O_3.

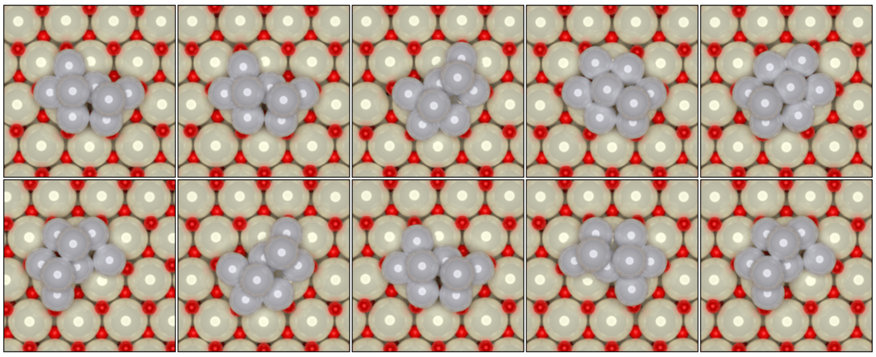

Figure S14: Geometries of the 10 lowest-energy clusters obtained through ANN-driven GA searches for Pt_8/CeO_2.

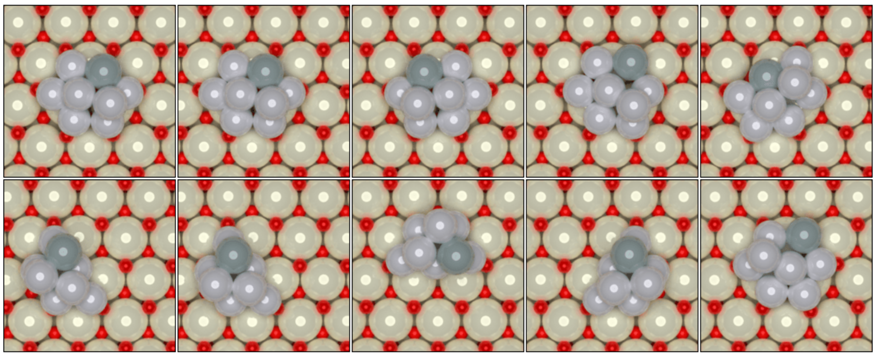

Figure S15: Geometries of the 10 lowest-energy clusters obtained through ANN-driven GA searches for Pt_7Sn_1/CeO_2.

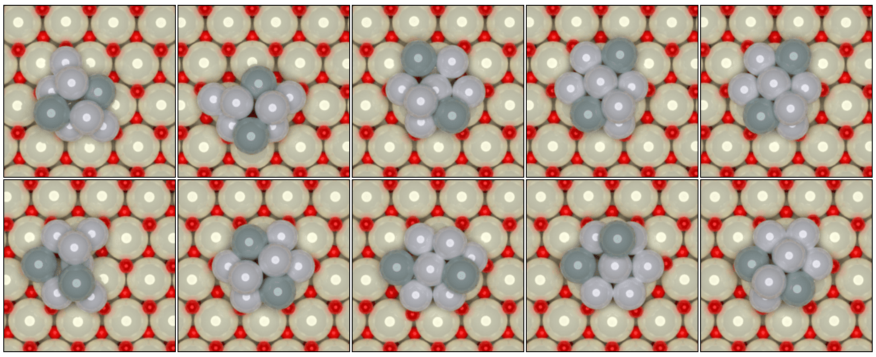

Figure S16: Geometries of the 10 lowest-energy clusters obtained through ANN-driven GA searches for Pt_6Sn_2/CeO_2.

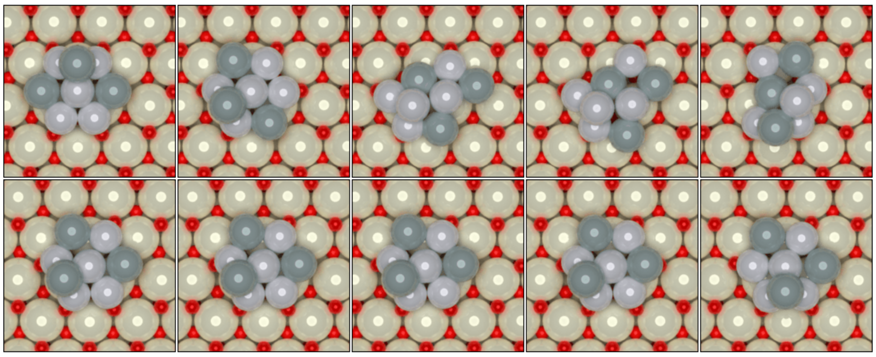

Figure S17: Geometries of the 10 lowest-energy clusters obtained through ANN-driven GA searches for Pt_5Sn_3/CeO_2.

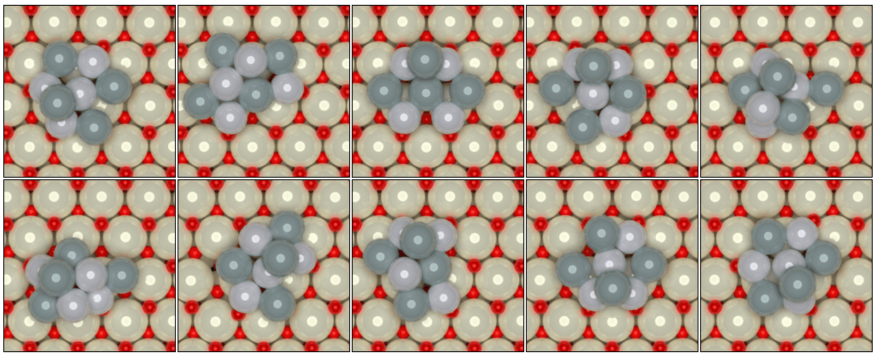

Figure S18: Geometries of the 10 lowest-energy clusters obtained through ANN-driven GA searches for Pt_4Sn_4/CeO_2.

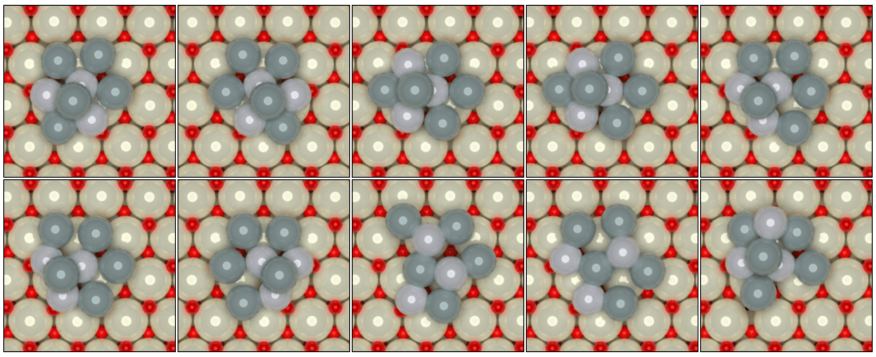

Figure S19: Geometries of the 10 lowest-energy clusters obtained through ANN-driven GA searches for Pt_3Sn_5/CeO_2.

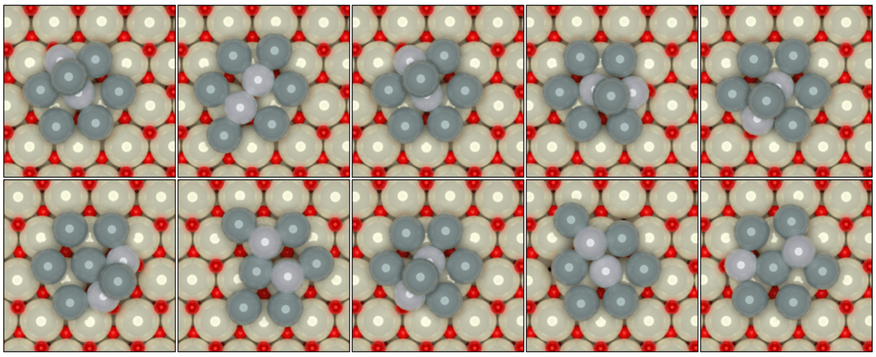

Figure S20: Geometries of the 10 lowest-energy clusters obtained through ANN-driven GA searches for Pt_2Sn_6/CeO_2.

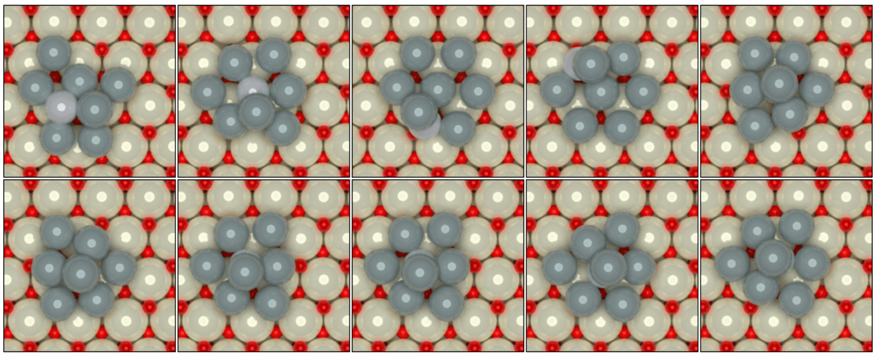

Figure S21: Geometries of the 10 lowest-energy clusters obtained through ANN-driven GA searches for Pt_1Sn_7/CeO_2.

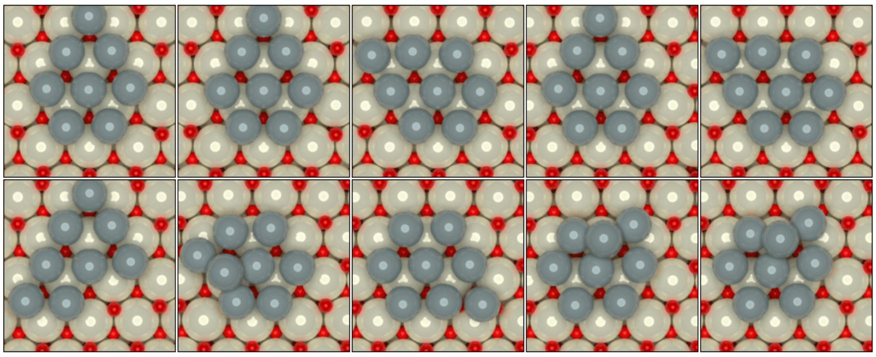

Figure S22: Geometries of the 10 lowest-energy clusters obtained through ANN-driven GA searches for Sn_8/CeO_2.

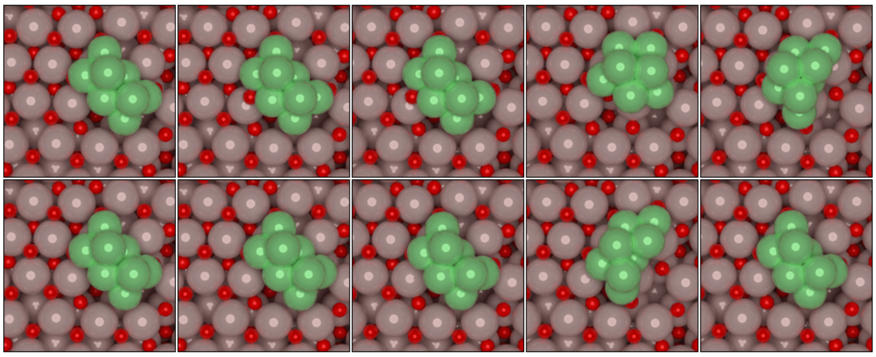

Figure S23: Geometries of the 10 lowest-energy clusters obtained through ANN-driven GA searches for Ni_8/In_2O_3.

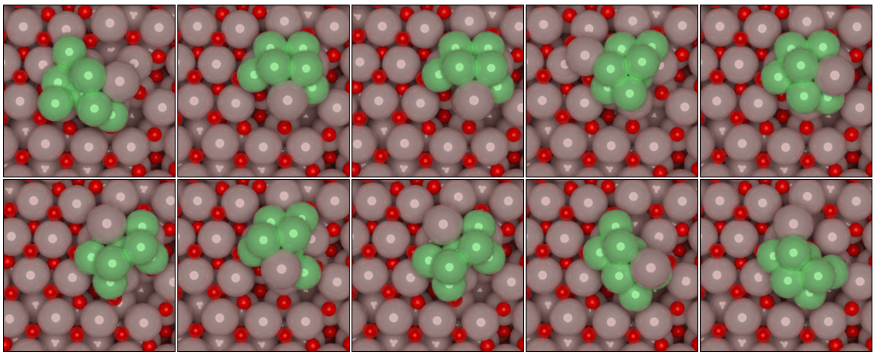

Figure S24: Geometries of the 10 lowest-energy clusters obtained through ANN-driven GA searches for Ni_7In_1/In_2O_3.

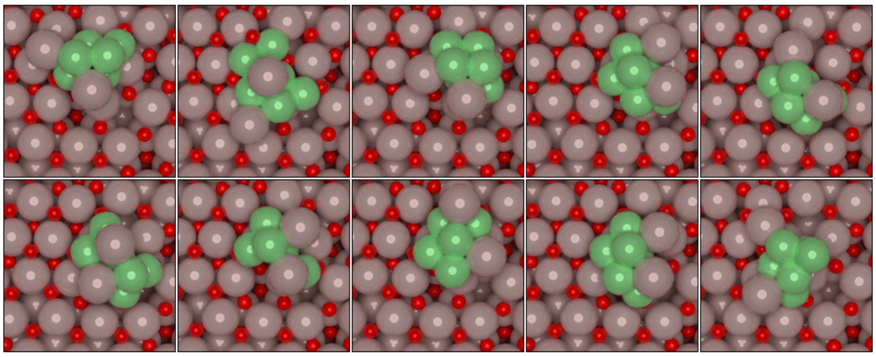

Figure S25: Geometries of the 10 lowest-energy clusters obtained through ANN-driven GA searches for Ni_6In_2/In_2O_3.

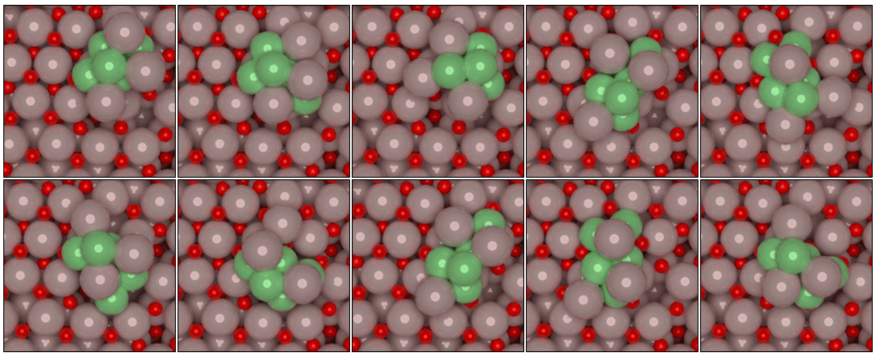

Figure S26: Geometries of the 10 lowest-energy clusters obtained through ANN-driven GA searches for Ni_5In_3/In_2O_3.

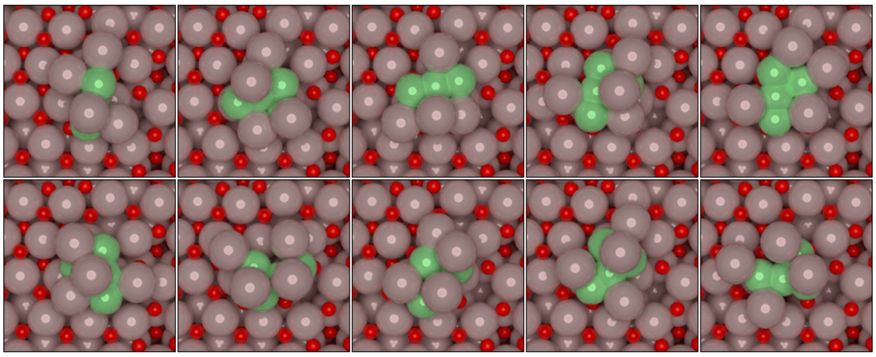

Figure S27: Geometries of the 10 lowest-energy clusters obtained through ANN-driven GA searches for Ni_4In_4/In_2O_3.

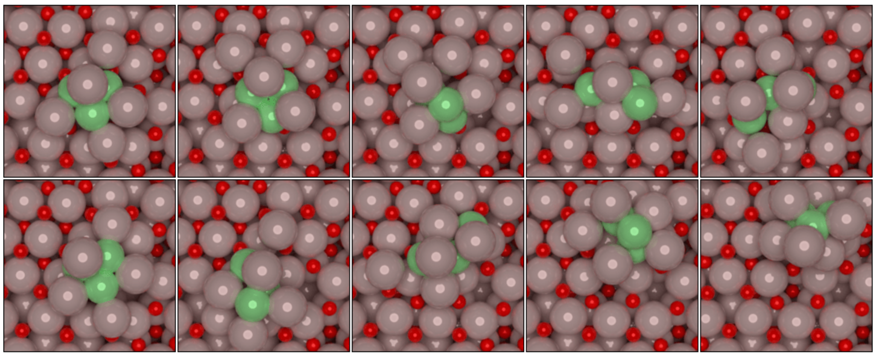

Figure S28: Geometries of the 10 lowest-energy clusters obtained through ANN-driven GA searches for Ni_3In_5/In_2O_3.

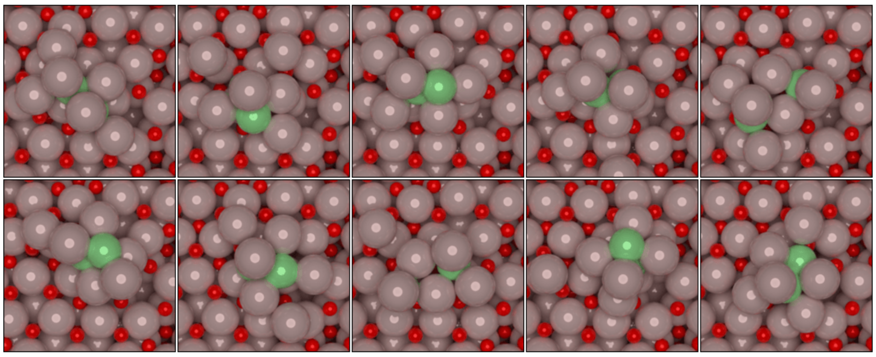

Figure S29: Geometries of the 10 lowest-energy clusters obtained through ANN-driven GA searches for Ni_2In_6/In_2O_3.

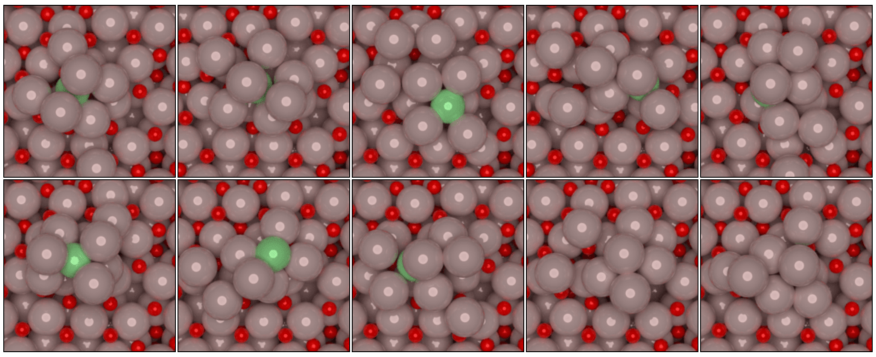

Figure S30: Geometries of the 10 lowest-energy clusters obtained through ANN-driven GA searches for Ni_1In_7/In_2O_3.

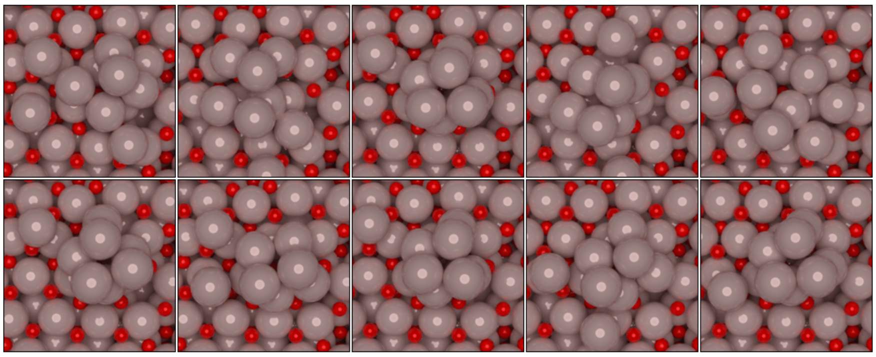

Figure S31: Geometries of the 10 lowest-energy clusters obtained through ANN-driven GA searches for In_8/In_2O_3.

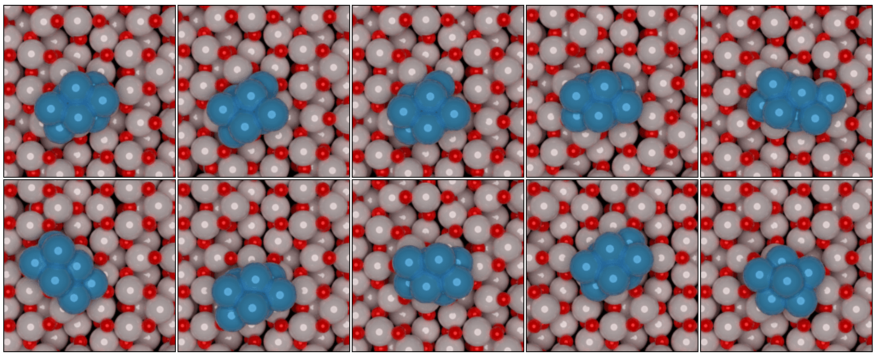

Figure S32: Geometries of the 10 lowest-energy clusters obtained through ANN-driven GA searches for Co_8/Al_2O_3.

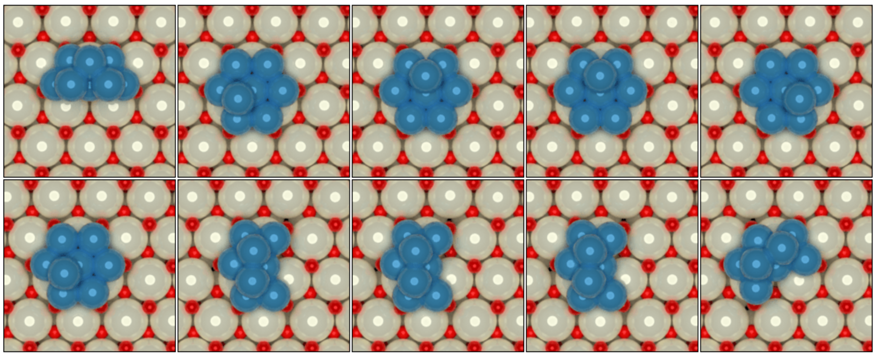

Figure S33: Geometries of the 10 lowest-energy clusters obtained through ANN-driven GA searches for Co_8/CeO_2.

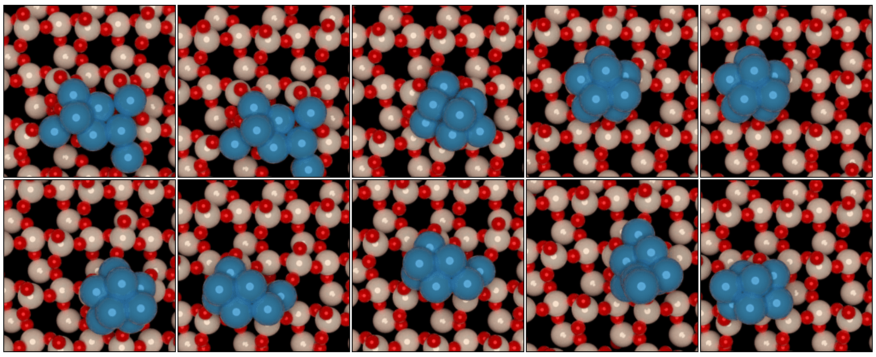

Figure S34: Geometries of the 10 lowest-energy clusters obtained through ANN-driven GA searches for Co_8/SiO_2.

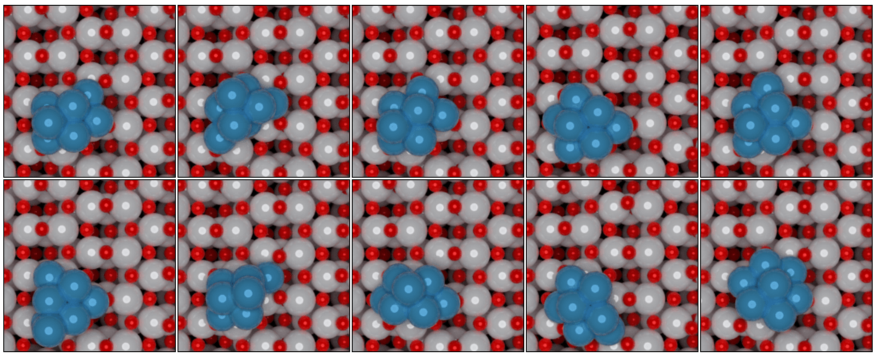

Figure S35: Geometries of the 10 lowest-energy clusters obtained through ANN-driven GA searches for Co_8/Ant-TiO_2.

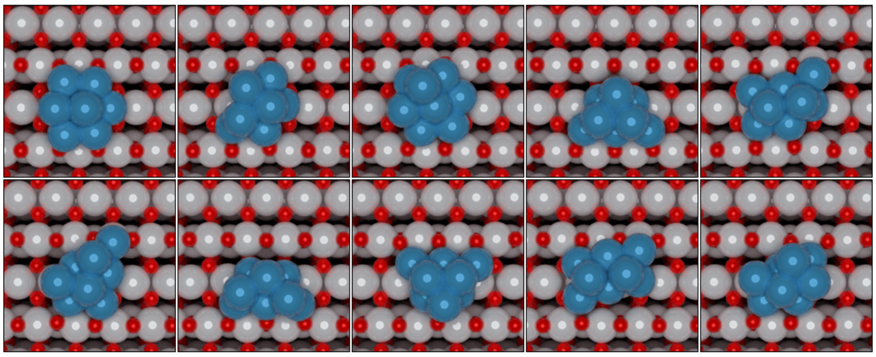

Figure S36: Geometries of the 10 lowest-energy clusters obtained through ANN-driven GA searches for Co_8/Rt-TiO_2.

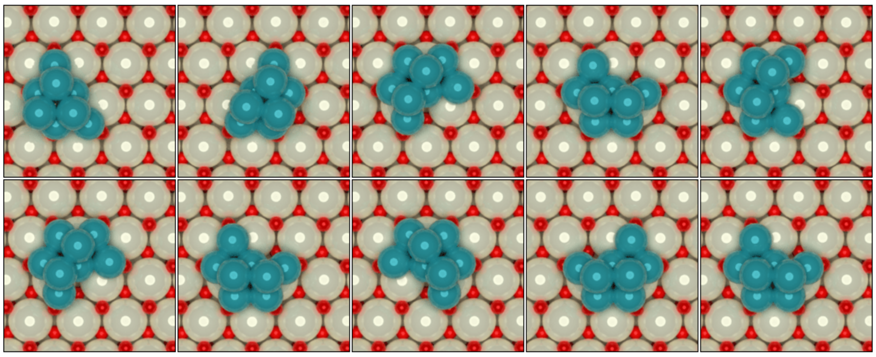

Figure S37: Geometries of the 10 lowest-energy clusters obtained through ANN-driven GA searches for Rh_8/CeO_2.

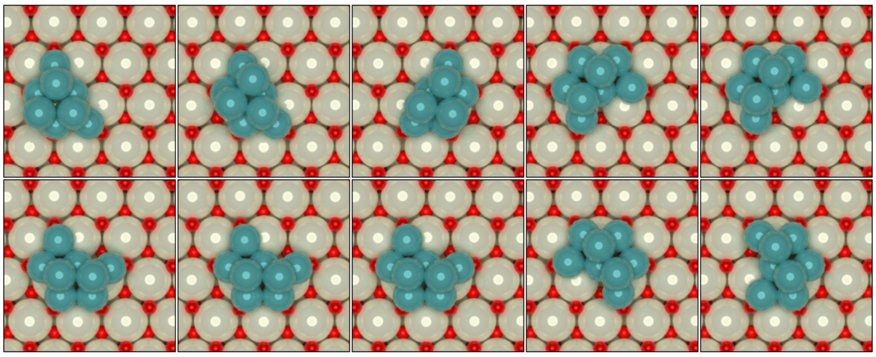

Figure S38: Geometries of the 10 lowest-energy clusters obtained through ANN-driven GA searches for Ru_8/CeO_2.

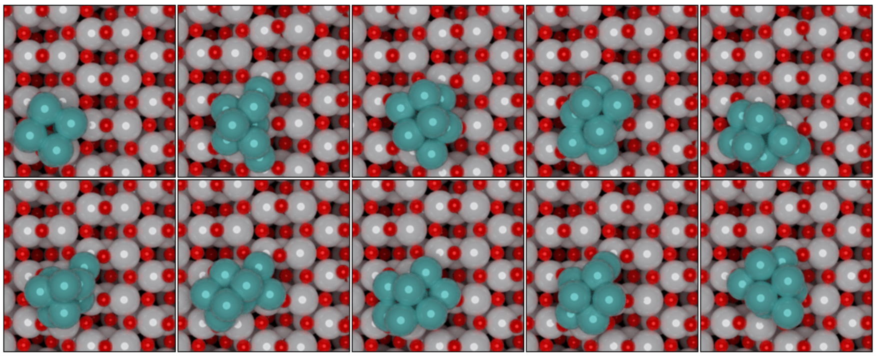

Figure S39: Geometries of the 10 lowest-energy clusters obtained through ANN-driven GA searches for Ru_8/Ant-TiO_2.

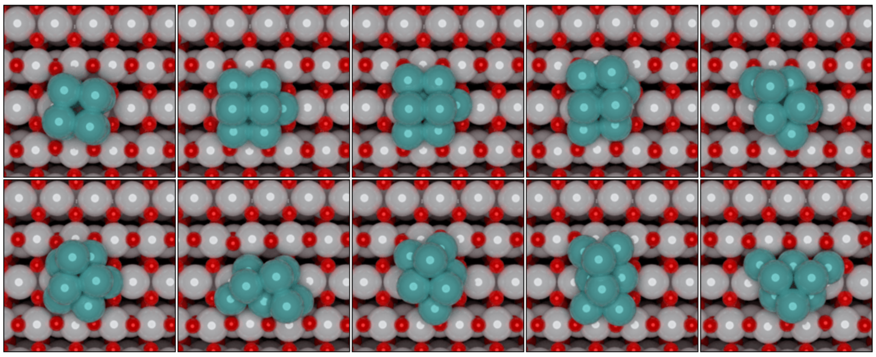

Figure S40: Geometries of the 10 lowest-energy clusters obtained through ANN-driven GA searches for Ru_8/Rt-TiO_2.

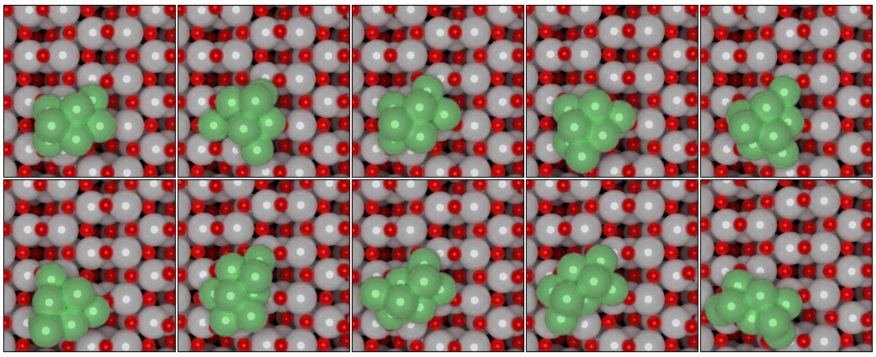

Figure S41: Geometries of the 10 lowest-energy clusters obtained through ANN-driven GA searches for Ni_8/Ant-TiO_2.

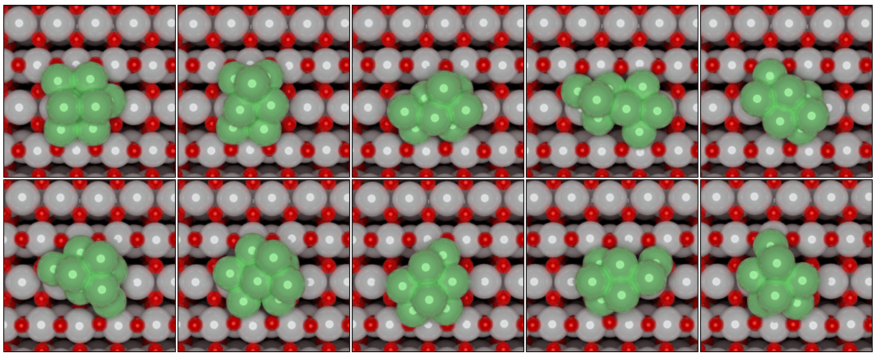

Figure S42: Geometries of the 10 lowest-energy clusters obtained through ANN-driven GA searches for Ni_8/Rt-TiO_2.

4 DFT-ANN LEME Comparisons

In order to quantify the difference in cluster coordinates predicted by DFT and ANN, the Frobenius norm of the difference matrix is computed.

$$||F|| = \sqrt{\sum_{i}^{N} ||\bar{r}^{\mathrm{ANN}} - \bar{r}^{\mathrm{DFT}}||^2} \tag{S9}$$

$\bar{r}$ denotes the coordinate vector of each atom in the cluster. Inter-cluster atomic ordering is determined through sequential minimisation of interatomic distances to account for indexing differences between samples.

Because the clusters in the LEME sets differ between DFT and ANN predictions, mapping of clusters is performed using CSR matching as detailed earlier. The corresponding indices are reported alongside the norms.

Table S13: Comparison of LEME cluster geometries from GA searches using DFT and ANN. Coordinates are compared using the Frobenius norm of the difference matrix. Values are reported in Å. Inter-cluster atomic ordering is determined through sequential minimisation of interatomic distances. I denotes the index of the cluster in the LEME sets.

	Pt_8		Pt_6Sn_2		Pt_4Sn_4		Pt_2Sn_6		Sn_8	
I_{DFT}	I_{ANN}	$\|\|F\|\|$	I_{ANN}	$\|\|F\|\|$	I_{ANN}	$\|\|F\|\|$	I_{ANN}	$\|\|F\|\|$	I_{ANN}	$\|\|F\|\|$
0	0	0.039	0	0.074	0	0.012	2	0.025	1	0.139
1	1	0.016	1	0.018	1	0.113	3	0.010	2	0.045
2	2	0.161	2	0.096	2	0.062	4	0.043	3	0.175
3	3	0.160	3	0.045	4	0.156	5	0.009	4	0.038
4	4	0.150	5	0.034	6	0.117	6	0.160	5	0.099
5	5	0.066	6	0.044	7	0.035	7	0.094	6	0.012
6	6	0.149	7	0.149	8	0.128	8	0.066	7	0.093
7	7	0.125	9	0.079	9	0.071	9	0.092	8	0.087
8	10	0.020	10	0.075	11	0.115	10	0.041	9	0.104
9	11	0.004	11	0.132	12	0.004	11	0.059	10	0.048

5 Unsupported Co Clusters

A DFT-driven GA was performed to initialise a transfer dataset on unsupported Co_8 clusters. This dataset was extended to clusters of 6 to 20 atoms through ANN-driven GA initialised on geometries obtained from geometric adatom adsorption. Results are reported in Figure S43. Cost reduction of the transfer method compared to DFT-driven GA searches is similar to that of the SiO_2-supported clusters. The cost of the DFT-driven GA for larger clusters increases more rapidly compared to the supported systems. This is attributed to the lack of a templating effect by the support, enhancing sample diversity. As a result, cost reduction from the ANN increases as particles grow larger.

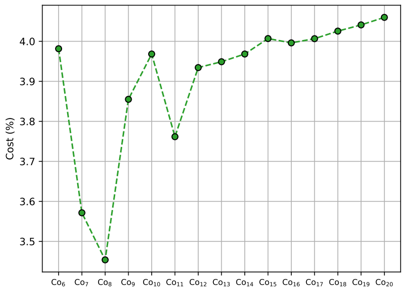

Figure S43: Relative cost of the transfer dataset method for GA searches of unsupported Co nanoclusters compared to DFT-driven GA. ANN were initialised using structures obtained through adatom adsorption along a geometric potential. Median values are reported. The cost of DFT post-optimisations is not included.

References

[1] B. Klumpers, E.J.M. Hensen, I.A.W. Filot, Lateral Interactions of Dynamic Adlayer Structures from Artificial Neural Networks, *J. Phys. Chem. C* **2022**, *126*, 5529–5540

[2] X. Glorot, Y. Bengio, Understanding the Difficulty of Training Deep Feedforward Neural Networks, *Proceedings of the Thirteenth International Conference on Artificial Intelligence and Statistics*, Journal of Machine Learning Research: Workshop and Conference Proceedings, vol. 9, ISSN: 1938-7228, **2010**, 249–256
